# Born to Cry: A Genetic Dissection of Infant Vocalization

**DOI:** 10.3389/fnbeh.2018.00250

**Published:** 2018-10-29

**Authors:** David George Ashbrook, Snigdha Roy, Brittany G. Clifford, Tobias Riede, Maria Luisa Scattoni, Detlef H. Heck, Lu Lu, Robert W. Williams

**Affiliations:** ^1^Department of Genetics, Genomics and Informatics, University of Tennessee Health Science Center, Memphis, TN, United States; ^2^Department of Physiology, College of Veterinary Medicine, Midwestern University, Glendale, AZ, United States; ^3^Research Coordination and Support Service, Istituto Superiore di Sanità, Rome, Italy; ^4^Department of Anatomy and Neurobiology, University of Tennessee Health Science Center, Memphis, TN, United States

**Keywords:** infant ultrasonic vocalizations, quantitative trait loci, heritability, parent-of-origin effects, crying, genetic dissection, BXD, USV

## Abstract

Infant vocalizations are one of the most fundamental and innate forms of behavior throughout avian and mammalian orders. They have a critical role in motivating parental care and contribute significantly to fitness and reproductive success. Dysregulation of these vocalizations has been reported to predict risk of central nervous system pathologies such as hypoxia, meningitis, or autism spectrum disorder. Here, we have used the expanded BXD family of mice, and a diallel cross between DBA/2J and C57BL/6J parental strains, to begin the process of genetically dissecting the numerous facets of infant vocalizations. We calculate heritability, estimate the role of parent-of-origin effects, and identify novel quantitative trait loci (QTLs) that control ultrasonic vocalizations (USVs) on postnatal days 7, 8, and 9; a stage that closely matches human infants at birth. Heritability estimates for the number and frequency of calls are low, suggesting that these traits are under high selective pressure. In contrast, duration and amplitude of calls have higher heritabilities, indicating lower selection, or their importance for kin recognition. We find suggestive evidence that amplitude of infant calls is dependent on the maternal genotype, independent of shared genetic variants. Finally, we identify two loci on Chrs 2 and 14 influencing call frequency, and a third locus on Chr 8 influencing the amplitude of vocalizations. All three loci contain strong candidate genes that merit further analysis. Understanding the genetic control of infant vocalizations is not just important for understanding the evolution of parent–offspring interactions, but also in understanding the earliest innate behaviors, the development of parent–offspring relations, and the early identification of behavioral abnormalities.

## Introduction

Vocal communication is important for social interactions in all mammals, and it begins in rodents on the first postnatal day with the communication between an infant and its parents (Doty, 1974; Ehret and Haack, 1982; Maggio and Whitney, 1986; Oller et al., 2013; Arriaga, 2014). Mouse pups produce high frequency ultrasonic vocalizations (USVs) when stressed by loss of body temperature (Okon, 1970a), by hunger or by separation from the mother (Noirot, 1966; Ehret, 2005). Pups also vocalize in response to intense tactile stimulation (Okon, 1970b). These USVs are critical in triggering parental attention (Noirot, 1964; Smith, 1976; Ehret and Haack, 1982; Cohen-Salmon et al., 1985; D’Amato and Populin, 1987; Hernandez-Miranda et al., 2017) and to solicit food (Branchi et al., 2001). Abnormalities in infant crying patterns or acoustic characteristics can have diagnostic value. For example, the cries of a human baby have been reported to sound different during a benign colic or to predict risk of central nervous system pathologies such as hypoxia, meningitis, or more recently autism spectrum disorder (Sheinkopf et al., 2012; Gelfand, 2016; Chittora and Patil, 2017; Esposito et al., 2017; Smarius et al., 2017). Rodent USVs are prominent soon after birth and are therefore useful as a phenotyping tool (Roubertoux et al., 1996; Scattoni et al., 2009; Roy et al., 2012; Wöhr, 2014; Riede et al., 2015).

Differences in infant vocalization are prominent among genotypes and strains of mice and their intercrosses (Bell et al., 1972; Cohen-Salmon et al., 1985; Hahn et al., 1987, 1997; Roubertoux et al., 1996), but no common DNA sequence variants underlying differences in this innate form of communication have yet been mapped in humans or any other vertebrate. Here, we have used the expanded BXD family of mice to identify novel, naturally occurring gene loci that control several important facets of infant vocalizations. The BXD family consists of ∼150 strains generated by crossing C57BL/6J (B6) and DBA/2J (D2) strains of mice, and then inbreeding their progeny for more than 20 generations to produce so-called recombinant inbred strains (Peirce et al., 2004). Infant vocalizations of the parents of the BXD family differ significantly for a number of quantitative traits (Bell et al., 1972; Hennessy et al., 1980; Cohen-Salmon et al., 1985; Roubertoux et al., 1996; Hahn et al., 1997; Thornton et al., 2005). As shown in the present study, this leads to a wide phenotypic range among the BXD progeny.

The BXD family is widely used for mapping QTLs that influence behavioral, anatomical, and physiological phenotypes (Carneiro et al., 2009; Rosen et al., 2009; Philip et al., 2010; Xue et al., 2015; Delprato et al., 2017). The BXDs are particularly useful for developmental analyses of behavior because each strain and genome can be studied at many time points and using nearly isogenic sets of males and females. This makes it highly practical to study gene-by-environment interactions, sex differences, and indirect genetic effects (IGEs; Ashbrook et al., 2015a, 2017; Ashbrook and Hager, 2017; Baud et al., 2017). We have previously shown that IGEs of the offspring genome influence maternal behavior (Ashbrook et al., 2015a, 2017; Ashbrook and Hager, 2017), and differences in infant vocalizations may be a key factor controlling parental care.

Acoustic characteristics such as fundamental frequency and sound amplitude, have different effects on parental care (Lingle et al., 2012), and we therefore expect that these characteristics have different heritabilities (Gustafsson, 1986; Price and Schluter, 1991; Branchi et al., 2001; Thornton et al., 2005). A low heritability usually indicates intense neutralizing selection, whereas high heritabilities are often associated with phenotypes that either do not greatly affect fitness (relaxed selection) or that are controlled by balancing selection (Walsh and Lynch, 2018) that increases variability, perhaps in order to enhance kin and litter recognition (Forstmeier et al., 2009; Blumstein et al., 2013). Hahn et al. (1997) hypothesized that rates of calling has undergone more intense selection than other characters (Hahn et al., 1997), however Spence et al. (2016) have shown that acoustic characteristics usually do not change in isolation but are highly interrelated (Spence et al., 2016). Here, we have expanded the analysis of USVs in order to comprehensively evaluate heritability of spectral, temporal, and sound amplitude characteristics. We have categorized and quantified a series of distinct call categories—also known as the syllabic patterns (Scattoni et al., 2008a)—for genetic analysis. We combine these two methods to estimate heritability for both quantitative and qualitative characteristics of infant vocalizations. We expand on this using a morphological analysis of the vocal organ in order to identify potential sources of acoustic variation.

In this study, we have used a diallel cross of C57BL/6J (B6) and DBA/2J (D2) to estimate heritability and parent-of-origin effects of all USV traits. We follow up with a larger study using 41 member strains of the BXD family, to map loci for both the frequency and amplitude of calls. We ask four questions:

• Can we detect parent-of-origin effects, such as parental genetic effects (PGEs), contributing to infant USVs?
• How are the functions of different aspects of vocalization reflected in their heritability?
• What regions of the genome modulate different facets of USVs, and are these different facets under selective or joint control?
• Within these genomic regions, can we identify putative candidate genes with mechanisms that link to different aspects of infant vocalizations?

## Materials and Methods

**FIGURE 1:**
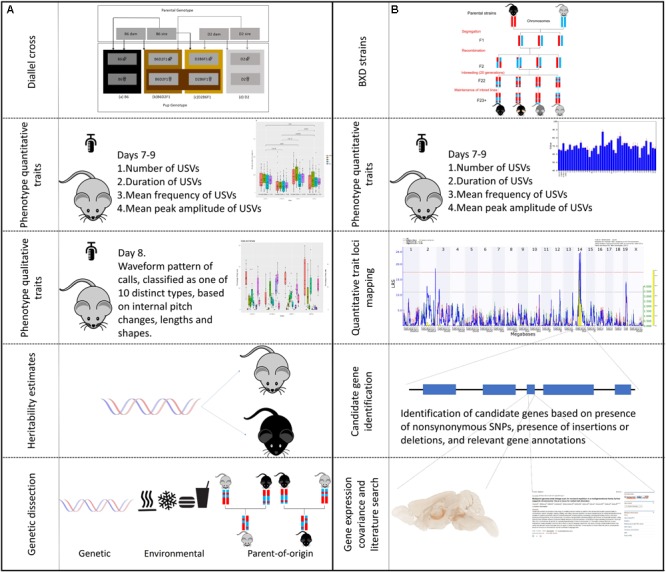
Outline of experimental design. The study is separated into two, complementary, parts, one using the BXD family for trait mapping, and the other using the parental diallel cross to study heritability and qualitative traits. The workflow of different aspects of the experiments are shown. **(A)** BXD strains were derived by crossing C57BL/6J (B6) and DBA/2J (D2) parents. The resulting heterozygous F1 mice were crossed to produce non-reproduceable F2 offspring representing recombinations of the parental B6 and D2 genomes. These F2 offspring were then inbred by sib-mating for 20 generations, at which point each strain represents a reproducible, unique mosaic of B and D alleles. These strains can then be maintained for repeated study of genetically identical individuals. Using a subset of 41 BXD strains we measured four quantitative traits (duration, amplitude, frequency, and number of calls) on postnatal days 7, 8, and 9. The traits were then mapped using GeneNetwork to identify significant quantitative trait loci (QTLs). The candidate genes within these QTLs were then selected based on the presents of non-synonymous single nucleotide polymorphisms (nsSNPs) or insertions/deletions (InDels). These candidate genes were then examined using gene annotation databases, gene expression data and literature searches. **(B)** A diallel cross of the two parental (B6 and D2) strains was produced, to create B6D2F1 and D2B6F1 offspring. These offspring are genetically autosomally identical except for which parent each allele was inherited from. The same four quantitative traits as above were measured in these animals, as well as the waveform pattern of their vocalizations on day 8, to produce 10 qualitative traits. Heritability was estimated, and dissected into the relative contribution of genetics, environment and parent-of-origin effects.

There were two major components of this study, estimation of heritability and parent-of-origin effects by a diallel cross, and identification of QTLs using the BXD family (Figure 1). For the first part of the study, we studied a diallel cross of four strains of mice using six litters each: C57BL/6J (B6) (*n* = 29), DBA/2J (D2) (*n* = 24), and reciprocal F1 hybrids—B6D2F1 (*n* = 30) and D2B6F1 (*n* = 30). The four genotypes of pups consisted of: (a) B6 (B6 dam and B6 sire), (b) D2 (D2 dam and D2 sire), (c) B6D2F1 (B6 dam and D2 sire), and (d) D2B6F1 (D2 dam and B6 sire). For the second part of the study we collected USV data for 41 strains of the BXD recombinant inbred family (*n* > 310). This family is the most deeply phenotyped mammalian model system, consisting of experimentally tractable and genetically defined mouse strains capturing a large amount of naturally genetic variation, analogous to that found in a normal human population (Wang et al., 2016). BXD strains were chosen pseudo-randomly dependent on availability, as no previous data was available for USVs in this population. We used BXD strains 1 (4 litters), 9 (6 litters), 24 (4 litters), 29 (4 litters), 32 (4 litters), 34 (4 litters), 40 (8 litters), 43 (4 litters), 44 (8 litters), 45 (6 litters), 48 (4 litters), 48a (8 litters), 49 (4 litters), 50 (8 litters), 51 (6 litters), 55 (4 litters), 60 (4 litters), 61 (4 litters), 62 (6 litters), 64 (4 litters), 65a (6 litters), 68 (4 litters), 69 (4 litters), 70 (6 litters), 71 (6 litters), 73 (4 litters), 73b (4 litters), 74 (6 litters), 75 (4 litters), 77 (4 litters), 83 (4 litters), 84 (4 litters), 85 (4 litters), 86 (4 litters), 87 (8 litters), 89 (6 litters), 90 (8 litters), 98 (9 litters), 99 (4 litters), 100 (4 litters), and 102 (6 litters). Strains 43–102 were generated using an advanced intercross (Peirce et al., 2004), and there is clear evidence for different levels of relatedness among these strains (Simecek et al., 2017). It is therefore helpful to adjust for kinship when mapping, as described below.

Parents and pups were raised in a closed-barrier specific-pathogen-free vivarium at the University of Tennessee Health Science Center (Nash Annex). Infants were weighed and recorded at postnatal day (P)7, P8, and P9, between 2 and 5 PM (CST). Postnatal days 7–9 were chosen as they closely correspond to full term birth in humans. This was estimated using the Translating Time website^1^ based on Workman et al. (2013), where mouse post-conception day 30 (approximately postnatal day 9) translates to human post-conception day 280 (approximately full term birth). This work was performed following guidelines approved by the UTHSC Animal Care and Use Committee.

### Ultrasonic Vocalization Measurement

Individual pups were separated from their parents and placed into a freshly cleaned plastic container (12 cm × 8 cm × 7 cm) that was located in the center of large polystyrene foam box (28 cm × 21 cm × 18 cm). The end of an UltraSoundGate 116–200 microphone—sensitive to frequencies up to 340 kHz—was placed 8–10 cm above the pup and the temperature within the box was ∼21°C. Vocalizations were recorded for the first 3 min of isolation at a sampling rate of 300 kHz using 16-bit A-to-D converter (Avisoft Bioacoustics ^2^). Prior to the end of each session, body temperature was measured on the chest using an infrared thermometer (IR-B153 from Braintree Scientific, Inc. ^3^). Recordings were transferred to Avisoft-SAS Lab Pro (version 4.40), and sonograms over the range of 20–250 kHz were generated using the following software parameters: (1) FFT length of 1,024 points (an interval of 4 ms), (2) frame size of 100%, (3) a Hamming smoother with a bandwidth of 317 Hz and a resolution of 244 Hz, (4) temporal resolution 1.024 ms with a Hamming smoother overlap of 75%, and 1/bandwidth of 3.2 ms.

### Quantitative Analysis of Vocalizations

Quantitative analysis of vocalization was carried out for: (1) the number of calls, (2) the duration of calls, (3) the mean fundamental frequency, and (4) the mean peak amplitude of calls. These parameters were measured automatically using Avisoft-SAS Lab Pro software for the entire 3-min recording session on P7 to P9. Automated measurements excluded calls with insufficient signal-to-noise ratio. SAS-lab Pro allows the investigator to overlay spectrographic images and fundamental frequency tracking results. The accuracy of call detection by the software was verified manually by an experienced user. The user verified all the calls in all recorded files and, when necessary, missed calls were marked by hand to be included in the automatic parameter analysis. With regard to peak amplitude measurement, this was considered as the point with the highest energy within the spectrum of the call, and was recorded as the relative output voltage of the microphone signal. Due to the consistent placement of the microphone, some clipping of high-volume calls was noted, but this did not correlate with strain, and therefore will simple result in reduced estimates of amplitude. Peak frequency was defined as the frequency at the location of the peak amplitude within the spectrum of the call. All measurements were carried out using the same equipment in the same chamber. No differences in quantitative parameters of calling were detected in a comparison of sexes; therefore, data was collapsed. This is in line with previous findings of no differences between sexes (Hahn et al., 1997). Average values of all parameters were also calculated for each case over the 3 days of testing and used for the statistical comparison.

### Qualitative Analysis of Vocalizations

Waveform patterns of calls recorded on P8 in D2, B6, B6D2F1, and D2B6F1 mice were examined in detail. Every call was classified as one of 10 distinct types (representative images in Supplementary Figure 1), based on internal pitch changes, lengths, and shapes, following the USV call categorization method proposed by Scattoni et al. (2008a). Each genotype produced 10 sonogram files from five different litters. We utilized one male and one female per litter. Sonograms were 3 min in length and selected from recordings at P8. We classified a total of 700 calls from B6, 1,305 from D2, 2,047 from B6D2F1 calls, and 1,972 from D2B6F1 calls. Average values of all call categories were used for the statistical comparison of D2, B6, B6D2F1, and D2B6F1 genotypes.

### Morphology of the Vocal Organs

Ultrasonic vocalizations are generated in the larynx by a whistle mechanism (Roberts, 1975; Riede, 2011). A specific laryngeal morphology facilitates the whistle (Riede et al., 2017), and laryngeal and respiratory movements determine spectral, temporal, and amplitude characteristics (Riede, 2011, 2013). If acoustic variation is caused by morphological differences of the vocal organ, we expect this to be reflected in one or more of the following characteristics: size of laryngeal cartilages (cricoid, thyroid and arytenoid cartilages), and the size of intrinsic larynx muscles (thyroarytenoid, cricothyroid, and lateral cricoarytenoid muscle). The size of these laryngeal structures were estimated through volume estimations using area measurements in serial histological sections. Additionally, we measured body mass, femur length, skull length, and skull width in order to estimate overall body size.

Specimens were fixed in formalin for 3 days and decalcified for 8 h. Body mass, skull width and length and femur length were measured on each pup. The head-neck region was embedded in paraffin. Coronal serial sections (5-μm-thick) were taken every 50 microns starting ventral. Sections were stained with hematoxylin–eosin for a general overview. Sections were scanned with ScanScope CS2 (Aperio, Vista, CA, United States). Volume of cricoid cartilage, thyroid cartilage, arytenoid cartilage, thyroarytenoid muscle (TA), lateral crico-arytenoid muscle (LCA), and cricothyroid muscle (CT) were estimated from digital images of sections from 10 equally distant levels from ventral to dorsal. All measurements were made using ImageJ (vers. 1.47; NIH). Volumes were estimated by adding up the products of area measurements (mm^2^) and the distance (in mm) to the next section level.

### Statistical Analyses

Statistical analyses of the diallel cross were performed using R. ANOVA was used to analyze both qualitative and quantitative USV measurements and to evaluate strain-dependent effects. Pairwise comparison between strains was done using Tukey *post hoc* analysis. The Kruskal–Wallis test was used to analyze body weight differences and USV traits between groups.

Heritability was estimated as the fraction of variance explained by strains in a simple ANOVA model. Sex was included in the initial analysis of vocal traits, but was not a useful predictor, and was not used as a cofactor. To compute an average estimate of heritability for each trait across all 3 days, we weighted the four heritability values by the inverse square of the errors. To calculate empirical confidence intervals, we used R to repeatedly recalculated heritability using 100,000 subsets of 95% of the data (e.g., for the day 7 traits in the BXD ∼295 of 310 samples) and recorded the 95th percentile of these as our 95% confidence intervals.

Different heritability values are given for the diallel cross as it is possible to deconvolute additive, dominance and parent-of-origin effects on heritability, and therefore we can report a narrow sense heritability (*h*^2^). In the inbred BXD, offspring and parents are genetically identical, and therefore we cannot differentiate different components of heritability, and instead get a single, broad sense, heritability (*H*^2^).

We also calculated $H_{\mathrm{RI}\bar{x}}^{2}$ (Belknap, 1998), the heritability of strain means, in contrast to the heritability for individuals. This is defined as $H_{\mathrm{RI}\bar{x}}^{2}$ = *V*_a_/(*V*_a_ + *V*_e_/*n*), where *V*_a_ is the genetic variability (variability between strains), *V*_e_ is the environmental variability (variability within strains), and *n* is the number of within line replicates.

### Genetic Dissection of Pup Vocalization

Density plots were produced in R using the *density* function in the *stats* package and plotted using the *ggplot2* package (Wickham, 2016). Values were converted to *z*-scores, to aid in the comparison between plots. Normal probability plots, a special case of the q–q plot, were produced in GeneNetwork, and can be reproduced there.

### QTL Mapping

For QTL mapping, the average of each vocalization trait was calculated for each strain. Distributions of these means were generally close to normal and did not require scale transformations (Supplementary Figure 6). However, a few means were high outliers, and these values (>+ 2.5 z) were winsorized (shifting values so as to approximate a more normal distribution; Shete et al., 2004). In all cases, we have explicitly entered both the original and the winsorized values in trait descriptions in www.genenetwork.org, so that interested readers can evaluate impact (for example, see BXD Phenotype ID: 16359, ‘Central nervous system, behavior, development, communication: Infant vocalization, mean peak frequency of call on day 7, mixed sexes from 2 to 5 litters [kHz] (Data winsorized, BXD86 from 55.419 to 56.192)’). All mean strain data are publicly available at GeneNetwork.org (GN phenotype identifiers: 16355–16362, 16600–16603, 16610–16612, 16617–16621, 18459–18461).

We initially mapped QTLs using the fast linear regression equations of Haley and Knott (1992). Genome-wide significant (*p* < 0.05), and suggestive (*p* < 0.63) thresholds were calculated by 5,000 permutations of the phenotypes, using GeneNetwork. The suggestive threshold corresponds to approximately one false QTL per genome-scan. Likelihood ratio statistic (LRS) scores were converted to log of the odds (LODs) scores by dividing by 4.61, and confidence intervals were defined by a LOD drop of 1.5 on either side of the peak value (Manichaikul et al., 2006). We considered QTL intervals that achieved genome-wide significance for one phenotype, and genome-wide suggestive for others, as highest priority for candidate gene analysis.

The January 2017 BXD genotype file was used ^4^.

Updated linear mixed model mapping algorithms are now available on GeneNetwork 2^5^ (Sloan et al., 2016), that account for kinship among strains. These new algorithms include GEMMA (Zhou and Stephens, 2012), pyLMM ^6^ (Sul et al., 2016), and R/qtl2^7^. Traits with significant and suggestive QTLs detected using simple interval mapping were remapped using GEMMA to confirm LRS scores and locations of QTLs even in the presence of kinship structure.

### Candidate Gene Identification

We examined genes within the QTL 1.5 LOD confidence intervals using QTLminer (Alberts and Schughart, 2010). We focused on genes with non-synonymous single nucleotide polymorphisms (nsSNPs) or insertions/deletions (InDels). We treated these genes as higher priority candidates, although we acknowledge that causal variants will often lie in a non-coding region. For each of these high priority candidates we then examined which Gene Ontology biological processes (Ashburner et al., 2000; Gene Ontology Consortium, 2015) and KEGG pathways (Kanehisa and Goto, 2000; Kanehisa et al., 2012) the gene was annotated as being part of, and highlighted those which may relate to vocalization. We also reviewed known effects of mutations in these high priority candidates using the Mouse Genome Informatics (MGI) *Phenotypes, Alleles and Disease Models Search* ^8^ (Bello et al., 2015). To cover more recent findings, which may not yet be reflected in annotation databases, we also checked the *NCBI Entrez* information ^9^ about the gene and its human homolog, particularly *Related articles in PubMed* and *GeneRIFs*.

We also examined expression of these candidate genes using gene expression databases. Gene expression data in P4 B6 mice is available from the *Allen Brain Atlas, Developing Mouse Map* (Allen Institute for Brain Science, 2008; Sunkin et al., 2013), and this was used to confirm where in the whole brain these genes are expressed. To get data specific to the BXD family, transcriptome data for many different organs and tissues are available in GeneNetwork, including some tissues with data at different ages. We surveyed all brain regions included in GeneNetwork (P3 neocortex, P3 striatum, P14 neocortex, P14 striatum, adult amygdala, adult cerebellum, adult hippocampal precursor cells, adult hippocampus, adult hypothalamus, adult midbrain, adult neocortex, adult nucleus accumbens, adult prefrontal cortex, adult striatum, adult ventral tegmental area) to find which of these transcripts have *cis*-eQTLs, and the expression of which correlate with USV traits. Both support the hypothesis that variant in these genes may be influencing vocalization phenotypes.

## Results

### Diallel Cross

#### Quantitative USV Traits

**FIGURE 2:**
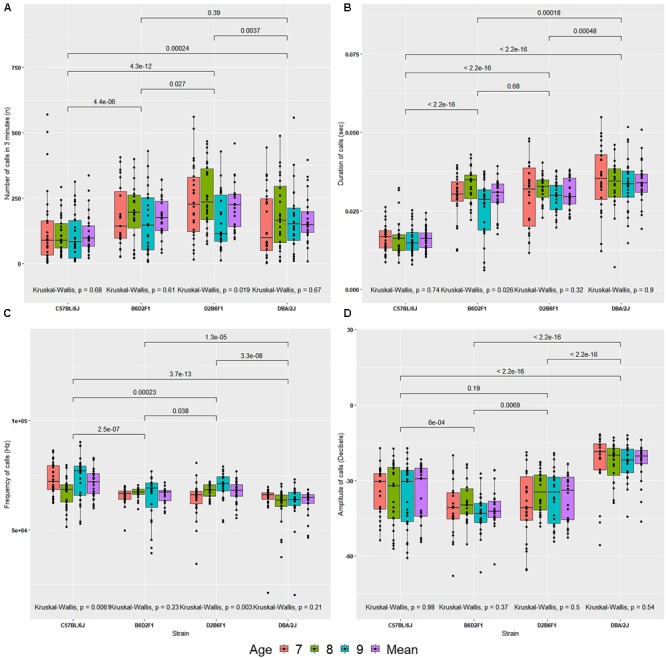
Values for four quantitative traits in the diallel cross **(A–D)** on postnatal days 7, 8, and 9, and the mean across days. **(A)** Number of calls, **(B)** duration of calls, **(C)** peak frequency of calls, **(D)** peak amplitude of calls. The bars above the graph show the significance of Wilcoxon test comparisons between the strains. Kruskal–Wallis significance values are shown below the graph for comparisons between age within each strain.

The number of USVs made over a 3-min interval varied by strain (Figure 2A). Averaged across the 3 days of observation, D2 pups made more calls than B6 pups [170 ± 19 (SEM) versus 114 ± 14, *p* < 0.0005]. This is in line with previous observations (Roubertoux et al., 1996). F1 hybrids made more calls than B6 and D2 (B6D2F1 = 179 ± 16 and D2B6F1 = 219 ± 18; *p* < 0.01, apart from DBA/2J and B6D2F1 where there was not a significant difference). Using all four cofactors and their interactions (sex, body weight, age and litter identifier), the *F* ratio associated with strain was 26.913, and while this value was highly significant (*p* < 2E-12, 3 df) the corresponding estimate of narrow sense heritability (*h^2^*) was modest—approximately 0.11. Litter identifiers can be used to estimate parental effects and these effects are strong (*F* = 10.335, *p* < 1E-14, 18 df) explaining 25% of the variance. Neither body weight nor sex were a predictor over the 3-day recording interval, explaining < 1% variance (*p* > 0.05) each. Age did have a small, significant effect (*F* = 2.914, *p* < 0.04, 3 df), but only explains 1% of the variance (Supplementary Table 1).

Call duration differed significantly by strain (Figure 2B). D2 made longer USVs than B6 (D2 = 34 ± 1.4 ms, B6 = 16 ± 0.6 ms; *p* < 5E-16). Duration of calls did not differ between the reciprocal F1s (B6D2F1 = 29 ± 1.2 ms and D2B6F1 = 30 ± 1 ms; *p* > 0.5). The estimate *h^2^* was high: ∼0.51. Litter identifiers, used to estimate parental effects, were found to have moderate effects (*F* = 8.418, *p* < 2E-12, 18 df), explaining ∼10% of the variance. Again, neither age nor sex were predictors and explained < 1% of the variance (*p* > 0.5). As above, age has a small significant effect, but explains < 1% of the variance (Supplementary Table 1).

Peak frequency varied by strain [*F*(3,102) = 51.997, *p* < 2E-16, *h^2^* = 0.16; Figure 2C]. Mean frequency of calls emitted by B6 pups was higher than D2 pups (B6 = 71.37 ± 1.18 kHz, D2 = 62.48 ± 1.23 kHz, *p <* 0.00001). But frequency produced by D2B6F1 and B6D2F1 pups did not differ significantly (mean frequency of call: B6D2F1 = 65.64 ± 0.83 kHz and D2B6F1 = 67.29 ± 1.01 kHz, *p >* 0.5). Neither sex, age or body weight had any significant direct effect. Again, litter effects were significant, explaining 23% of the variance (Supplementary Table 1).

Mean peak amplitude showed a significant difference by strain [*F*(3,102) = 304.633, *p* < 2E-16, *h^2^* = 0.31, Figure 2D]. B6 pups continuously emitted soft calls over all days whereas D2 pups called consistently louder (mean peak amplitude: B6 = –36 ± 2 dB, D2 = –23 ± 1.5 dB, *p* < 0.0000001). F1 pups produced low amplitude calls compared to B6 and D2 (B6D2F1 = -41 ± 1.6 dB and D2B6F1 = -37 ± 1.9 dB). Litter identifier explained 51% of the variance. Both sex and age had small, significant effects, but explain less than 1% of the variance (Supplementary Table 1).

Taken together, these results (summarized in Supplementary Table 1) show that all four quantitative traits have a strong heritable component (strain was significant for all of them), and therefore are appropriate for further genetic study in the BXD RI family. However, as we can resample the same genome repeatedly in inbred strains, the conventional heritability underestimates the heritability of the strain mean: that is, the heritability in a particular genome (which we can resample in inbred lines) rather than heritability in a particular individual (conventional heritability). This is referred to as $H_{\mathrm{RI}\bar{x}}^{2}$ (Belknap, 1998)_._ Our estimates of $H_{\mathrm{RI}\bar{x}}^{2}$ ranged from 0.62 for number of calls on day 9 to 0.98 for duration of calls on day 8 (Table 1).

**Table 1:** $H_{\mathrm{RI}\bar{x}}^{2}$ estimates (heritability accounting for repeated sampling of the same genome) for quantitative traits in the B6/D2 diallel cross, measured on postnatal days 7, 8, and 9.

Phenotype	Postnatal day	$H_{\mathrm{RI}\bar{x}}^{2}$
Number of call	7	0.85
Number of call	8	0.84
Number of call	9	0.62
Duration of calls	7	0.96
Duration of calls	8	0.98
Duration of calls	9	0.95
Frequency of calls	7	0.9
Frequency of calls	8	0.82
Frequency of calls	9	0.86
Amplitude of calls	7	0.97
Amplitude of calls	8	0.97
Amplitude of calls	9	0.96

*Values are from 29 C57BL/6J (B6), 24 DBA/2J (D2), 30 B6D2F1, and 30 D2B6F1 individuals, split into six litters of C57BL/6J (B6) and DBA/2J (D2) strains, and five litters of each of the F1 genotypes.*

#### Qualitative USV Traits

**FIGURE 3:**
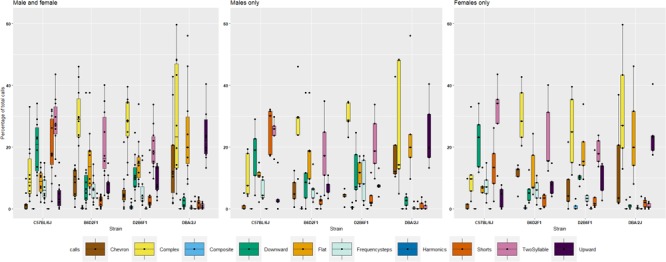
Percentage of ten different call types in the diallel cross on postnatal day 8, showing females, males and combined values by strain. Percentages were calculated in each genotype as number of calls in each category for each subject/total number of calls analyzed in each subject. Number of total calls analyzed: B6 700; D2 = 1305; B6D2F1 = 2047 and D2B6F1 = 1972. Calls were collected from 40 individuals, one male, one female, from five litters of each of the four genotypes.

Scattoni et al. (2008a) have defined ten distinct vocalization “syllables” generated by pups. To explore if these “syllables” show heritability, we performed an initial analysis on P8 in the diallel cross. All stains generated eight syllables; however, relative frequency of syllable types differs markedly among strains (Figure 3 and Supplementary Figure 1). D2 displayed high prevalence in production of complex (29%), flat (27%), upward (23%) and chevron (15%) calls, however, very low prevalence of downward (2%) and short (2%) calls on P8. In contrast, B6 pups produced two syllables (28%), short (20%), frequency steps (8%), flat (8%), downward (19%), complex (12%), and upward (4%) calls. Of note, very few chevron calls (1%) were produced. Reciprocal F1 pups emitted a spectrum of syllables, and the vast majority of calls produced by F1s were complex calls (30%). Surpassing the diversity of B6 and D2, F1s can produce downward (19%), two-syllable (18%), and frequency step (5%), as well as upward (9%), chevron (7%) and flat (16%) calls (Figure 3). For each call type, an ANOVA was performed to quantify the effect of strain, litter, body weight and their interactions and a Tukey *post hoc* was used to identify differences between strains (Supplementary Table 2). For seven types of calls (chevron, flat, frequency steps, harmonics, short, two-syllable, and upward) there was a significant effect of strain, and B6 and D2 were significantly different (Tukey *p* < 0.05) for all except harmonics, with only harmonics significantly different between B6D2F1 and D2B6F1 pups. Heritability was estimated for all qualitative traits and ranged from just 0.1 for harmonics to 0.65 for upward calls (Supplementary Table 2). We again also estimated $H_{\mathrm{RI}\bar{x}}^{2}$, which ranged from 0.57 for harmonics to 0.93 for upward calls (Table 2).

**Table 2:** $H_{\mathrm{RI}\bar{x}}^{2}$ estimates for qualitative ultrasonic vocalization traits in the B6/D2 diallel cross.

Qualitative phenotypes	$H_{\mathrm{RI}\bar{x}}^{2}$
Harmonics	0.57
Composite	0.68
Chevron	0.77
Frequency steps	0.77
Flat	0.78
Complex	0.84
Downward	0.87
Two syllable	0.88
Shorts	0.92
Upward	0.93

*These phenotypes were only measured on postnatal day 8. Values for 700 calls from B6, 1,305 from D2, 2,047 from B6D2F1 calls, and 1,972 from D2B6F1. Calls were collected from 40 individuals, one male, one female, from five litters of each of the four genotypes.*

#### Parent-of-Origin Effects on USVs

**FIGURE 4:**
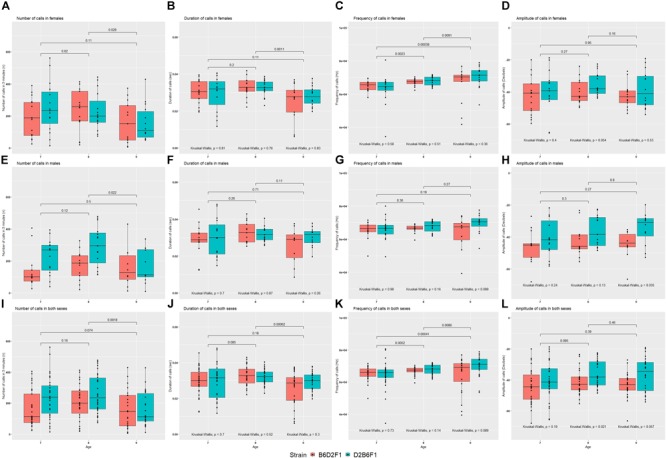
Mean values for the four quantitative traits in females **(A–D)**, males **(E–H)** and combined both sexes **(I–L)** of the reciprocal F1 cross (B6D2F1 and D2B6F1). Between day comparisons were calculated using the Wilcox test and are shown at the top of each plot, whereas comparisons between strains were calculated by the Kruskal–Wallis test and are shown at the bottom of each plot.

The reciprocal F1 females inherit identical nuclear genomes, including their X chromosomes, whereas males inherit identical autosomes, but differ in their sex chromosomes. For this reason, differences between the reciprocal F1 females are highly likely to be caused by either indirect parental effects or genomic imprinting, whereas differences between reciprocal F1 males could also be caused by the specific effects of the Y chromosome. Statistical comparison was performed on quantitative USV traits between reciprocal F1 pups, combined and split by sex (Figure 4). No significant difference was seen between reciprocal F1 females, although there was a suggestive difference in amplitude of calls on P8 (Kruskal–Wallis *p* = 0.054; Figure 4D). In males there were significant differences in number of calls on P7 and P8 (Figure 4E) and amplitude of calls on P9 (Figure 4H). In the combined data, number of calls on P7 (Figure 4I), and amplitude of calls on P8 (Figure 4L) were significantly different (Kruskal–Wallis *p* < 0.05).

#### Morphology of the Vocal Organs

**FIGURE 5:**
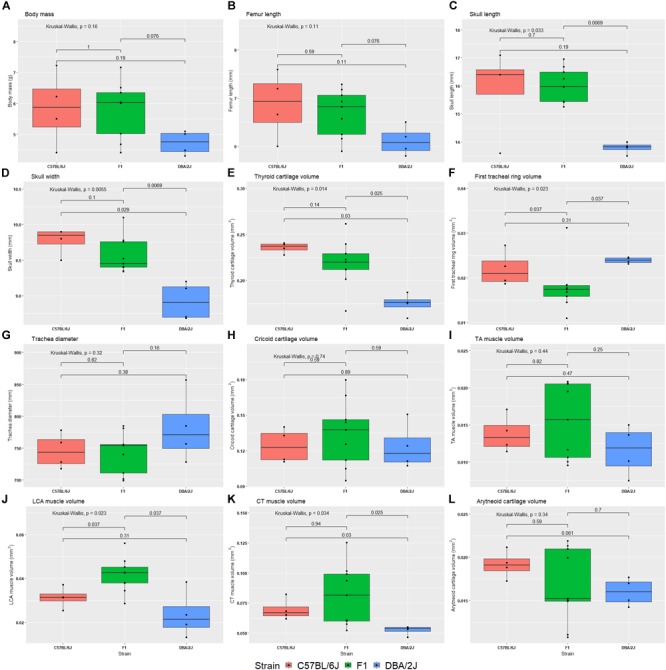
Box plots of 12 morphological phenotypes from the diallele cross. The reciprocal F1s are presented as a single group, because no significant difference was detected between the two for any of the morphological traits. The Kruskal–Wallis test was used to calculate the significance of the effect of strain (top left of each plot), whereas the Wilcox test was used to calculate pairwise differences between strains. Morphological phenotypes are body mass **(A)**, femur length **(B)**, skull length **(C)**, skull width **(D)**, thyroid cartilage volume **(E)**, first tracheal ring volume **(F)**, trachea diameter **(G)**, cricoid cartilage volume **(H)**, thyroarytenoid muscle volume **(I)**, lateral cricoarytenoid muscle volume **(J)**, cricothyroid muscle volume **(K)**, and arytenoid cartilage volume **(L)**.

We studied the effect of strain, age, sex, and their interactions on morphological characteristics by ANOVA, with Tukey Honest *post hoc* test. Between the reciprocal F1 strains we saw no differences in morphological characteristics. Therefore, these were combined, and the ANOVA analysis rerun. Body size is significantly influenced by strain (Table 3 and Figure 5A). Furthermore, thyroid cartilage size is significantly influenced by strain (Figure 5E).

**Table 3:** Estimated significance of strain, age, sex and their interactions on 12 quantitative morphological traits in the B6/D2 diallel cross, estimated by ANOVA.

Morphological phenotype	Strain	Age	Sex	Strain:Age	Strain:Sex	Age:Sex	Strain:Age:Sex
Body mass	**0.0116**	**0.0399**	0.2056	**0.0003**	0.1620	0.6681	0.8558
Femur length	**0.0187**	0.3237	0.8372	**0.0014**	0.1958	0.4186	0.3182
Skull length	**0.0002**	**0.0312**	0.9936	**0.0356**	**0.0102**	0.8402	**0.0431**
Skull width	**0.0049**	0.3792	0.4391	0.2621	0.8137	0.4446	0.8993
Thyroid cartilage volume	**0.0045**	0.0570	0.7983	0.1013	0.6366	0.3572	0.3717
First tracheal ring volume	0.0753	0.0585	0.5145	0.8565	0.4062	0.2107	0.1384
Trachea diameter	0.3390	0.8370	0.3540	0.6710	0.4390	0.3960	0.6030
Cricoid cartilage volume	0.5618	0.0862	0.1651	0.3255	0.3034	0.1747	0.4250
TA muscle volume	0.4420	0.6190	0.4000	0.3620	0.7690	0.2880	0.9080
LCA muscle volume	**0.0314**	0.4348	0.2033	0.4609	0.8954	0.8689	0.3682
CT muscle volume	0.1970	0.6280	0.8100	0.3530	0.6820	0.7880	0.6410
Arytenoid cartilage volume	0.5430	0.3900	0.8740	0.5010	0.7810	0.7430	0.3840

*Significant values (*p* ≤ 0.05) are shown in bold, suggestive values (*p* ≤ 0.1) are underlined.*

We also estimated heritability of morphological features using the same ANOVAs: Body mass *h*^2^ = 0.21, femur length *h*^2^ = 0.27, skull length *h*^2^ = 0.57, skull width *h*^2^ = 0.65, thyroid cartilage volume *h*^2^ = 0.58, first tracheal ring diameter *h*^2^ = 0.18, cricoid cartilage volume *h*^2^ = 0.058, TA muscle volume *h*^2^ = 0.14, LCA muscle volume *h*^2^ = 0.51, CT muscle volume *h*^2^ = 0.30, and arytenoid cartilage volume *h*^2^ = 0.12.

### Genetic Dissection of Pup Vocalization

Since we identified a clear effect of strain, and therefore of genotype, on quantitative USV phenotypes above (see the section “Quantitative USV Traits”), we can now examine whether these genetic effects are likely to be caused by a small number of loci with large effects, or a large number of loci of small effect.

**FIGURE 6:**
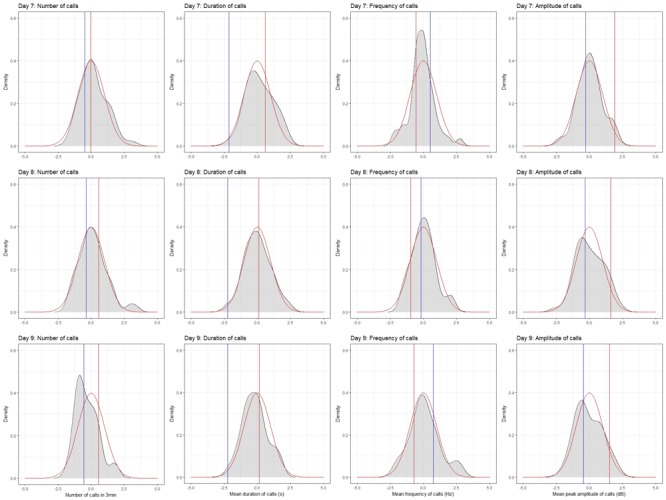
Density plots for the four quantitative traits in the BXD family on postnatal days 7, 8, and 9. Density plots were produced in R using the density function in the stats package and plotted using the ggplot2 package. Values were converted to *z*-scores, to aid in the comparison between plots. Density is shown in gray. The red line on each plot represents the normal distribution, whereas the red and blue bars represent the mean values for the D2 and B6 parents, respectively.

**FIGURE 7:**
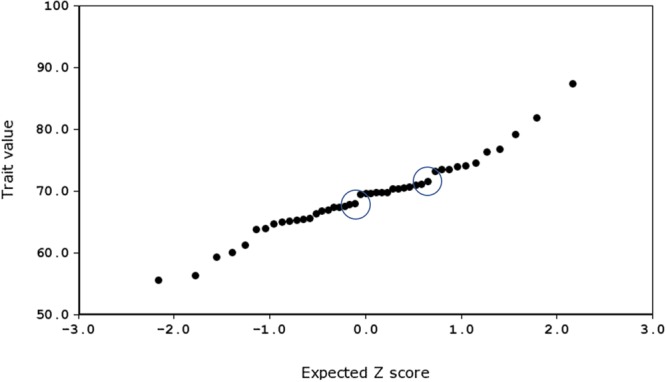
Normal probability plot for frequency of call on day 7, produced using GeneNetwork.org (Trait ID 16359). The two ‘jumps’ representing major effect loci are circled.

To do this, we produced probability density plots for all four quantitative traits, using the strain means and standard deviations of the mean for parents and all available BXD strains (Figure 6). These plots provide a visual assay of the complexity of the trait: at the extremes, a trait strongly modulated by one polymorphic locus would have two modes (representing the two homozygous genotypes—*BB* and *DD*), whereas more genetically complex traits modulated by many variants of small effects will be close to normal. Several of these plots appear to be made up of a mixture of two normal distributions (e.g., number of calls on day 7, amplitude on day 9), characterized by a ‘shoulder’ on the normal distribution, indicative of one locus of moderate to large effect segregating in the family. Other plots appear to be more normally distributed (e.g., duration of calls on day 8), suggesting several variants of smaller effect. Normal probability plots show the same patterns, with breaks (where the actual values suddenly jump, rather than following the x = y line) indicating a variant of large effect (e.g., the circled gaps in Figure 7, for call frequency on day 7). These are automatically generated in GeneNetwork. Normal probability plots for all 12 quantitative USV traits measured in the BXD population are shown in Supplementary Figure 6.

**FIGURE 8:**
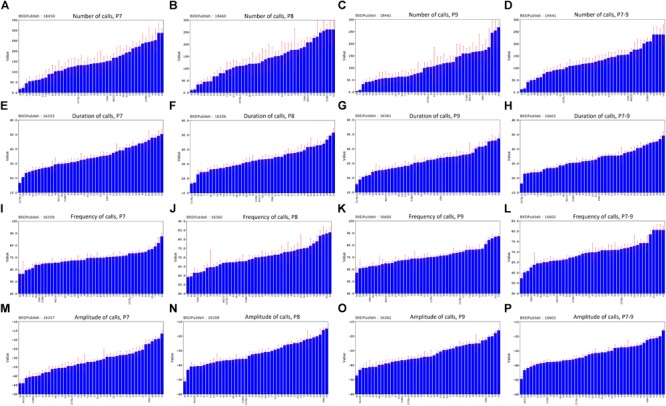
Distribution of mean values across the strains of the BXD family for the four quantitative traits, postnatal days (P)7–9 and combined P7–9. **(A–D)** Number of calls, **(E–H)** duration of calls, **(I–L)** frequency of calls, **(M–P)** amplitude of calls. BXDPublish is the trait ID in GeneNetwork, with which the trait can be reanalyzed.

The distribution of call duration among all strains was approximately continuous between 16 and 49 ms (Figure 8H). B6 pups produced the shortest duration calls among the BXD family on all 3 days (Figures 8E–H), whereas D2 pups were consistently among the top five strains for peak amplitude all 3 days (Figures 8M–P). Differences in the peak frequency are less consistent (Figures 8I–L) but the D2 strain was consistently in the bottom quartile. This could conceivably be an adaptation to impaired hearing of adult D2 mice (Cohen-Salmon et al., 1985; Zheng et al., 1999). The BXD family includes many strains with phenotypes more extreme that either B6 or D2 parent (Figures 8A–D).

#### Heritability

**FIGURE 9:**
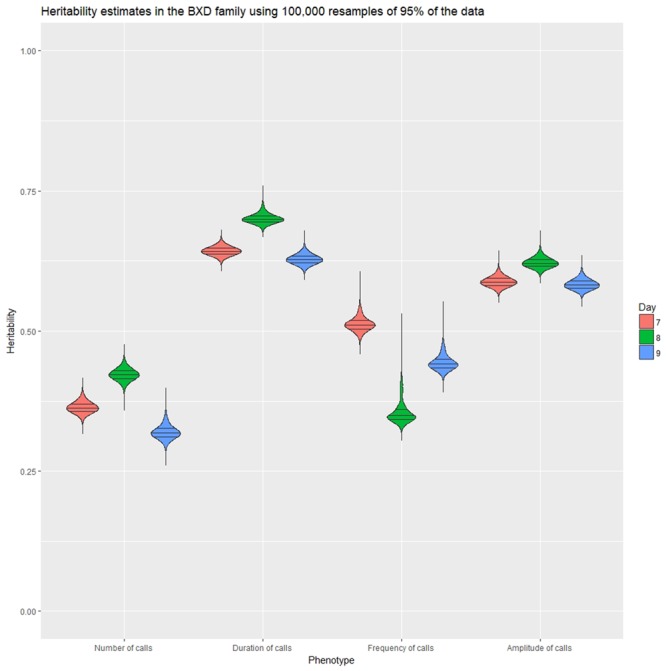
Violin plot of heritability estimates in 100,000 random subsamples 95% of the data for the four quantitative traits on postnatal days 7, 8, and 9. Quartile intervals are shown by black lines, and days are represented by red, green, and blue fill, respectively.

Heritability was estimated using a simple ANOVA model. On all three days, number and frequency of calls had the lowest heritability (*H*^2^ = 0.2–0.4) and duration and amplitude of calls had the highest heritability (*H*^2^ = 0.53–0.65), with relatively small confidence intervals (Table 4 and Figure 9). The heritability estimates in the BXD RI are higher than the narrow sense heritability estimates above for the diallel cross, however, the order of the heritabilities (duration and amplitude higher than number and frequency) is the same as the diallel cross. It is unsurprising that heritability estimates are higher in the RI, as the RI are composed of homozygotes, which contribute more to additive genetic variance than heterozygotes (Belknap et al., 1996; Belknap, 1998), and we calculated *H*^2^, which will include epistatic effects. Again, we calculated estimates for $H_{\mathrm{RI}\bar{x}}^{2}$, which ranged from 0.55 for number of calls on day 9 to 0.89 for peak amplitude on day 8 (Table 5), and they correlated well with estimates from the diallel cross (Spearman’s rho = 0.86, Supplementary Figure 7). Power calculations allow us to estimate our power to detect effects of a given effect size. With a $H_{\mathrm{RI}\bar{x}}^{2}$ value around 0.5, we can detect loci accounting for 0.5 of the genetic variance with 80% power (Supplementary Figure 8), while a $H_{\mathrm{RI}\bar{x}}^{2}$ of 0.9 gives us the power to detect loci account for ∼0.38 of the genetic variance with 80% power (Supplementary Figure 9).

**Table 4:** Heritability estimates for quantitative traits in the BXD population, measured on postnatal days 7, 8, and 9.

Phenotype	Postnatal day	*H*^2^ estimate	100,000 permutations, 95% data max *H*^2^	100,000 permutations, 95% data min *H*^2^
Number of calls	7	0.359	0.422	0.315
Number of calls	8	0.418	0.478	0.370
Number of calls	9	0.314	0.397	0.252
Duration of calls	7	0.640	0.684	0.609
Duration of calls	8	0.698	0.747	0.663
Duration of calls	9	0.625	0.677	0.590
Frequency of calls	7	0.507	0.621	0.450
Frequency of calls	8	0.347	0.535	0.302
Frequency of calls	9	0.438	0.555	0.394
Amplitude of calls	7	0.586	0.637	0.552
Amplitude of calls	8	0.619	0.682	0.587
Amplitude of calls	9	0.581	0.633	0.548

*Heritability was estimates using an ANOVA model, and this was repeated 100,000 times using random sub-samples of 95% of the data. Max heritability is the large heritability estimate in the 100,000 replicates and min heritability is the lowest heritability estimate in the 100,000 replicates.*

**Table 5:** $H_{\mathrm{RI}\bar{x}}^{2}$ (heritability accounting for repeated sampling of the same genome) estimates for quantitative ultrasonic vocalization traits in the BXD family, measured on postnatal days 7, 8, and 9.

Phenotypes	Postnatal day	$H_{\mathrm{RI}\bar{x}}^{2}$
Number of calls	7	0.7
Number of calls	8	0.77
Number of calls	9	0.55
Duration of calls	7	0.84
Duration of calls	8	0.88
Duration of calls	9	0.74
Frequency of calls	7	0.75
Frequency of calls	8	0.65
Frequency of calls	9	0.63
Amplitude of calls	7	0.82
Amplitude of calls	8	0.89
Amplitude of calls	9	0.78

*Up to 315 animals were used each day, from the 41 BXD strains, B6, D2, B6D2F1, and D2B6F1.*

#### QTL Mapping

Quantitative trait loci were mapped for number, duration, frequency, and amplitude of calls using Haley-Knott (HK) regression mapping (Supplementary Figures 2–5), PyLMM, and GEMMA as implemented in GeneNetwork. We found three QTLs which were genome-wide significant in at least one phenotype, and these were examined further to identify other phenotypes that had a genome-wide suggestive QTL at the same location.

**FIGURE 10:**
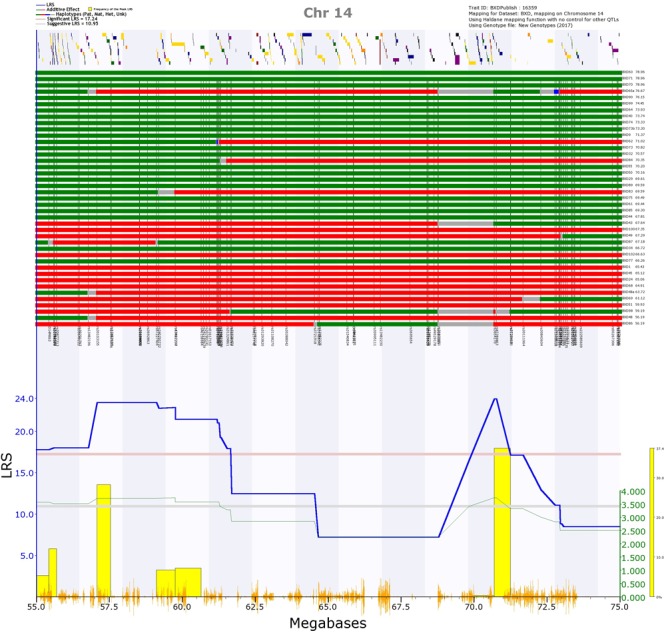
QTL map of the Chr 14 QTL for trait 16359, frequency of calls on postnatal day 7. In the top section, the location of genes are shown by yellow and purple blocks. Below this, the distribution of haplotype blocks in the 41 BXD strains is shown. Green represents the *D* allele, whereas red represents the *B* allele. It is clear that the majority of strains with high values (i.e., a high call frequency, right of the top section) have the *D* allele (green), whereas the majority of strains with the *B* allele (red) have low values (i.e., a low call frequency, right of the top section). In the bottom section, the blue line represents the genome scan, showing the likelihood ratio statistic (LRS) associated with each marker across the locus. The top, pink, line marks genome-wide significance (genome-wide *p* ≤ 0.05), the lower, gray, line the suggestive significance threshold (genome-wide *p* ≤ 0.63). The green line shows the additive coefficient, showing that the DBA/2J alleles increase trait values. The green axis on the right shows by how much the respective alleles increase trait values. The yellow bars represent the bootstrap values, with the left edge of each bar showing where the peak fell in each of the 2,000 resamples of the data.

The first was on Chr 14, and was significant for mean peak frequency on P7 (GN 16359; Supplementary Figure 4), and suggestive for mean peak frequency for all days combined (GN 16602; Supplementary Figure 4). When kinship was accounted for using GEMMA, this locus was also suggestive for mean peak frequency on P8 (GN 16360). The region appeared to consist of two QTLs: one spanning from 54.4 to 61.7 Mb, the other from 68.8 to 72.3 Mb (represented by two peaks rising above the significance threshold; Figure 10). Both of these positions appeared as significant with the GEMMA and pyLMM algorithms, suggesting they are not an artifact of the mapping method. Eight strains have different alleles at the two different peaks (that is, they have a *B* allele at the first peak and *D* allele at the second peak or a *D* allele at the first peak and a *B* allele at the second peak; Figure 10 upper rows). This suggests that the two peaks are two independent QTL, as it is not simply a continuation of the same allele with, for example, an unknown or heterozygous region between which reduces the LRS score. However, the two peaks have overlapping 1.5 LOD drop confidence intervals, meaning we cannot say with certainty whether they represent two loci or one. This longest potential confidence interval runs from 40.7 to 73.4 Mb. Having the *D* allele at this position increases the trait value (Figure 10; those strains with a green row at the top of the figure, representing the strain having the *D* allele at that position, tend to have a higher call frequency than those strains with a red row at the top of the figure, representing them having the *B* allele at that position).

The second QTL alters the same traits, and is located on Chr 2, with a remarkably short confidence interval from 179.5 to 180.8 Mb (Supplementary Figure 10). This locus is also confirmed using GEMMA and pyLMM. The *D* allele increases the trait value (strains with a *D* allele at this position tend to have a higher call frequency than those with the *B* allele at this position; Supplementary Figure 10).

The final QTL is on Chr 8, and is significant for peak amplitude on P7 (GN 16357), suggestive for peak amplitude for all days combined (GN 16603), and for peak amplitude on P8 (GN 16358; Supplementary Figure 5). The 1.5 LOD confidence interval is from 3.5 to 16.7 Mb (Supplementary Figure 11). As with the other two loci, the *D* allele increases trait values, and the QTL is again significant using GEMMA and pyLMM.

#### Candidate Gene Identification

For each of the QTLs identified above, all variants within the 1.5 LOD confidence interval were examined as potential candidate genes underlying the phenotype. The QTL on Chr 14 includes 407 genes, 237 of which have nsSNPs or InDels. Due to this large number, it is difficult to identify candidates with confidence, however, we highlight some genes of interest for future study in our Supplementary Material. For the remaining two QTL we were able to identify a small list of candidate genes.

The QTL on Chr 2 contains 44 genes, 31 of which have InDels or nsSNPs. These 31 genes are therefore candidates, as they contain variants which may affect their expression or function. To prioritize which of these 31 genes are most likely to be the gene underlying the phenotype we examined additional information: 8 of the 31 genes (*Ss18l1, Gtpbp5, Osbpl2, Lama5, 1600027N09Rik, Ogfr, Tcfl5, Dido1*) have a *cis*-eQTLs in transcriptome data, indicating that variants within the gene alter its expression; expression of 4 of these 31 genes (*Gtpbp5, Slco4a1, Ogfr, Slc17a9*) correlate well with at least one of the peak frequency phenotypes, which suggests that altering expression of that gene may alter expression of the phenotype; and 12 of the 31 genes (*Cdh4, Taf4, Psma7, Ss18l1, Osbpl2, Adrm1 (Rpn13), Lama5, Ntsr1, Col9a3, Dido1, Slc17a9, Bhlhe23*) are associated with brain, behavior, or cranio-facial morphology in previous datasets, e.g., in knockout animals, which suggests that the gene acts in tissues relevant to USVs (Supplementary Table 3). No gene appears in all three lists, and so it is difficult to narrow the list further from these 17 genes (i.e., 17 genes appear in at least one of the three lists).

The QTL on Chr 8 contains 185 genes, however, only 27 of these have InDels or nsSNPs. Therefore, we concentrate on these 27 genes as candidates and they were examined for the same information as the Chr 2 QTL above: 6 of the 27 genes (*Abhd13, Dlgap2, Arhgef10, 2900016B01Rik, Kbtbd11, Csmd1*) have a *cis*-eQTLs in transcriptome data; expression of 6 of these 27 genes (*Dlgap2, Arhgef10, 2900016B01Rik, Kbtbd11, Myom2, Csmd1*) correlate well with at least one of the mean peak amplitude phenotypes; and 11 of the 31 genes (*Fam155a, Abhd13, Col4a1, Col4a2, Arhgef7, Tfdp1, Dlgap2, Cln8, Arhgef10, Myom2, Csmd1*) are associated with brain, behavior, or cranio-facial morphology in previous datasets (Supplementary Table 4). *Dlgap2, Arhgef10*, and *Csmd1* appear in all three of these lists and are therefore are our mostly highly ranked candidates for altering mean peak amplitude of infant USVs. Further details of candidate genes can be found in the Supplementary Material.

## Discussion

Essentially all aspects of infant vocalization display striking and heritable differences. In this study, we have initiated a genetic dissection of these innate vocalizations with the ultimate goal of uncovering genes, sequence variants, and brain circuitry that underlie critical and innate behaviors. We computed heritability for 14 facets of vocalizations at a very early stage of development, equivalent to the perinatal period in humans (Workman et al., 2013) ^10^. We also evaluated the role of parent-of-origin effects on these behaviors. Our main findings are that the heritability for qualitative USV traits—the use of specific syllables —is generally higher than those of quantitative traits such as amplitude, duration, vocal rate, and fundamental frequency. However, the heritability of all traits was sufficiently high to warrant QTL analysis. Parent-of-origin effects were modest. Using a large cohort of BXD strains, we were able to map three QTLs for major quantitative features of infant vocalization to Chrs 2, 8, and 14.

### Heritability

We found that heritability varies significantly among quantitative and qualitative vocalization traits. Given the core function of these vocalizations in survival, it is probable that the underlying genetic mechanisms and CNS circuitry are conserved among mammals (Moore et al., 1997; Lingle and Riede, 2014). Heritability is an estimate of the fraction of variation of a trait generated by genetic factors in a specific population and environment. Heritability should not be confused with biological importance, and in fact, many of the most vital traits have low heritability due to intense stabilizing selection (Price and Schluter, 1991). Crying is one of the most instinctive and innate behaviors and arises spontaneously, and unlike more plastic and learned behaviors, we have good reasons to suspect that infant vocalizations should be under strong stabilizing selection and consequently to have low heritability.

Heritability of quantitative USV traits are indeed low: from 0.1 for vocal rate, 0.16 for fundamental frequency range, 0.30 for amplitude, to 0.5 for duration (Supplementary Table 1). Our results are concordant with previous work that focused on some of these traits (Hahn et al., 1997; Thornton et al., 2005) with heritabilities ranging from 0.1 to 0.35. The agreement is impressive despite methodological differences. We analyzed both quantitative and qualitative USV traits using a significantly longer sample period (3 min versus 18 s) and did not stimulate pups by cold temperatures or rough handling. The low heritability of call rate suggests that this attribute is under the strongest stabilizing selection.

In contrast, the higher heritability for call duration implies that this trait accommodates to greater variation without affecting growth and, ultimately, reproductive fitness. It would be of interest to determine if this finding generalizes to wild populations. Variation in syllabic call patterns also has a relatively high heritability and may not affecting viability. However, call syllabic pattern may play a role for kin recognition and quality of the maternal behavior (Brunelli et al., 2015), and there is evidence that vocalizations play an important function in the mutual recognition of mothers and offspring (Mogi et al., 2017). Mice show communal nesting behavior (Heiderstadt et al., 2014; Weidt et al., 2014), and there is a fitness advantage to being able to distinguish one’s own offspring in a communal nest. In the extreme case of Mexican free-tailed bat, mothers can recognize calls reliably in crèches with up to 4,000 pups per square meter (McCracken and Gustin, 2010).

We show that certain vocalization parameters, such as rate, are likely to be under strong stabilizing selection. This has been previously observed in a study of zebra finch, in which Forstmeier et al. (2009) demonstrated that heritability of non-learned calls produced by females was considerably higher (0.4) than those of learned songs produced by males (0.1), indicating strong stabilizing selection for male song (Forstmeier et al., 2009).

### Parent-of-Origin Effects

Parent-of-origin effects are defined as persistent differences in phenotypes of offspring caused by differences attributable strictly to parental genotype or phenotype. Reciprocal B6D2F1 and D2B6F1 female pups are genetically identical, including their X chromosomes. Only their mitochondrial genomes differ. However, the polarities of parents are reversed (Figure 1). Differences between reciprocal F1 female pups are therefore almost entirely due to parent-of-origin effects, genomic imprinting, or sampling error. Using our diallel cross design we have been able to detect these potential parent-of-origin effects, and detect a potential effect on peak amplitude of call.

Traits in one individual may be influenced by gene variants of other individuals, and these are referred to as social genetic effects or IGEs. The most obvious source of IGEs are PGEs, whereby the genotype of parents influences the phenotype of offspring. Siblings and other conspecific, and even parasites, pathogens, and predictors can be a strong source of IGEs (Baud et al., 2017). In contrast, genomic imprinting is due to epigenetic changes within the individual causing differential gene expression characterized by either complete or partial silencing of one parental allele (Barlow, 2011; Abramowitz and Bartolomei, 2012; Ashbrook and Hager, 2013). As both mothers and fathers had contact with the pups in our study, our observed PGEs could come from either parent.

Among quantitative USV traits only peak amplitude of call displayed a possible parent-of-origin effect. For call number, call duration, mean peak frequency, and all morphological traits, there were no significant parent-of-origin effect in reciprocal F1 females. In contrast, Thornton et al. (2005), detected maternal influences on rate, frequency, and duration of calls. The relative contributions of genetic background and early environmental factors on calling behavior in C57BL/6JOlaHsd and C57BL/6NCrl were studied by using an embryo-transfer procedure (Wöhr et al., 2008). Among several traits—call number, total calling time, call duration, peak frequency, peak amplitude, and frequency modulation—only amplitude was dependent on the genotype of the mother (Wöhr et al., 2008), in agreement with our findings.

The parental B6 and D2 mouse strains differ in their adult anxiety-related behavior. D2 has higher basal anxiety-like behavior than B6 (Mozhui et al., 2010). Differences in parental anxiety-related behavior may contribute to PGEs. A hypothetical anxiety-related parental effect in modulation of peak amplitude is of interest because it has been demonstrated that call amplitude can be reduced by anxiolytic drugs (Insel et al., 1986).

### Sources of Acoustic Variation

We found differences between B6 and D2 strains in four important acoustic features: (a) mean fundamental frequency is higher in B6, (b) sound amplitude is higher in D2 USVs, (c) ultrasonic calls are longer in D2 and (d) D2 pups vocalize at a higher rate (Figure 2). Which morphological or physiological characteristics could cause these differences? Our morphological results indicate that the B6 pups are larger in overall size than D2. Body mass, femur length, and skull dimensions are larger in the B6 (Figure 5). We know that USVs are produced by a laryngeal whistle mechanism, but larger body size does not necessarily translate to lower fundamental frequency. In order to produce an ultrasonic whistle, the glottal airflow interacts with the edge of the entrance hole into a laryngeal airsac (“ventral pouch” hereafter) which is located downstream from the vocal folds (Riede et al., 2017). The ventral pouch is entirely housed inside the laryngeal lumen surrounded by the thyroid cartilage (Riede et al., 2017). The fundamental frequency of an ultrasonic call is determined by two variables, (a) the distance between vocal folds and the ventral pouch edge, and (b) lung pressure (Riede et al., 2017). Both variables are precisely controlled by rodents (Riede, 2013). The fundamental frequency differences between B6 and D2 are therefore most likely explained by either of three variables: (a) laryngeal size difference, (b) laryngeal control differences, and/or (c) by different breathing movements. The greater thyroid cartilage size in B6 (Figure 5) would suggest a larger vocal fold-ventral pouch distance and this should result in lower fundamental frequencies, however, average fundamental frequency is higher in B6. Our findings therefore suggest that the difference in fundamental frequency that we see is caused by breathing movements.

In mammals, sound amplitude is predominantly controlled by lung pressure and secondarily by laryngeal configuration as well as interactions between the laryngeal sound source and the vocal tract filter (Riede and Brown, 2013). In rodent USVs, increased lung pressure seems to be related to an increased sound intensity (Riede, 2011), and control of breathing is essential for infant vocalizations (Hernandez-Miranda et al., 2017).

Longer call durations and a higher vocal rate can only be explained by differences in breathing movement control during vocalization. Cries are produced during the exhalatory phase of breathing (Riede, 2013; Sirotin et al., 2014). Previous work has shown different breathing patterns between mouse strains, including the B6, but the relationship between breathing movements and vocalization frequency has not been fully elucidated (Yamauchi et al., 2008; Chai et al., 2011). A dystonic rat model shows a close association between an overall slower breathing rate and different vocal characteristics compared to wild-type (Riede et al., 2015). Interestingly, rat pups that were selectively bred for high call rates of isolation-induced vocalization, produced calls of significantly increased amplitude but those pup with lower vocal rates also produced calls shorter in duration (Spence et al., 2016), suggesting that these vocal parameters are highly interrelated. A detailed study of the relationship between lung pressure, glottal configuration and rodent USV is necessary, and future studies should include respiratory measurements in the B6 and D2 strain and their hybrids in order to understand how differences in breathing control may translate into vocal differences.

How much are differences in brain development associated with acoustic variation between mouse strains? The brain is not only essential for controlling the breathing characterizes described above, but also for the ability to vocalize in response to appropriate stimuli. Selective breeding for certain traits, for example high call rates, also alters functions of brain systems and emotional behaviors throughout life (Spence et al., 2016). Candidate brain areas include the cerebellum, which plays an important role in vocalization and articulation. In mice, the cerebellum has been shown to be critically involved in generation of USVs (Fujita et al., 2008; Riede et al., 2015) whereas, in humans, the cerebellum is important for speech and language (Vias and Dick, 2017). Another important area is the nucleus tractus solitarius in the hindbrain (Hernandez-Miranda et al., 2017), which has been shown to be essential for infant vocalizations in mice.

Morphological features were only measured in the diallel cross, and not in the BXD lines. However, future studies will be able to employ a battery of physiological methods to understand strain-specific vocal motor control (Hegoburu et al., 2011; Riede, 2011) and new imaging techniques to determine shape differences in the vocal organ (Riede et al., 2017). This highlights one advantage of using inbred lines such as the BXD—future phenotypic measurements can be combined and correlated with the phenotypes we have collected, without all phenotypes needing to be measured in one individual or cohort.

### Genetic Results

We identified three loci influencing call fundamental frequency and amplitude. Each covers a region of the genome containing from 44 to 407 protein-coding genes. The great majority of studies concerning the genetics of USVs have exploited gene knockouts, and knockouts of *FoxP2, Cadherin-6, Fmr1, Drd2*, and vasopressin receptor genes are already known to produce abnormal USVs (Shu et al., 2005; Scattoni et al., 2008a,b; Wang et al., 2008; Nakagawa et al., 2012; Roy et al., 2012; Curry et al., 2013). However, our data suggest that vocalization is a complex trait, influenced by many variants of small effect. Therefore, although knockout studies give us insight into potential mechanisms by which genes can influence infant USVs, they are likely to have a much larger effect that variants causing natural variation within a population, for example, the QTLs for adult male USVs recently mapped in the BXD family (Knoll et al., 2017). Ehret (2005) identified three gene categories associated with pup USVs: (1) those that contribute to the development of sensory and perceptual systems (input), (2) those involved in regulation of emotion and motivation (processing), and (3) those linked to respiration and laryngeal control (output). Consequently, we concentrated on candidate genes known to be involved in (1) the sensory system, especially hearing, (2) neuronal development and morphology and (3) cranio-facial development and morphology. To find some of the natural variants underlying infant USVs, we performed QTL analysis on infant traits in 41 BXD strains. We mapped number, amplitude, frequency and rate of USVs on postnatal days (P)7, P8, and P9. We detected three QTLs which were significant for at least one of the traits, and found candidate genes within each which fell into the categories described above. We discuss some of these candidates in Supplementary Material.

### Limitations and Future Work

As with all experiments, there are limitations to our study which provide areas for future research and ongoing investigation. A significant limitation is that our qualitative analysis (i.e., syllabic structure) was only carried out on a single postnatal day. This was due to the significant manual labor that was necessary for this analysis by a highly trained investigator, which made the analysis low throughput. Therefore, any conclusions we draw from this qualitative analysis should not be generalized to represent the entire postnatal period. Future work will make use of new, high-throughput, less labor intensive systems, such as the MUPET-Mouse machine learning system (Van Segbroeck et al., 2017; Knoll et al., 2018). These new methods will not only allow us to expand the number of days analyzed, allowing a longitudinal analysis throughout early life, but also the number of strains. The BXD population now consists of over 150 strains, which would allow greater precision of mapping, and potentially > 22,000 different reciprocal crosses to map parent-of-origin effects. In light of this, our qualitative analysis is only a first step in a fuller investigation, expanding both longitudinally and laterally.

Similarly, our morphological analysis was only carried out in the diallel cross. This allowed us to demonstrate that morphological features which may be associated with vocalization can be detected and analyzed, but does not allow us to perform any genetic analyses. In future studied, we hope to be able to do this analysis in the BXD population, allowing direct correlation between our findings and morphological features, potentially detecting genomic variants underlying both.

Another large contributor to variance was litter effects. Litter effects encompass a large number of different aspects, from vivarium-related factors (e.g., position in the rack) to social effects (e.g., the total weight of the litter) to maternal effects (e.g., weight of the mother). It is therefore unsurprising that for some traits they can account for up to 51% of the variance (amplitude; Supplementary Table 1). These are all obviously interesting variables in themselves, and worthy of their own deeper investigations. However, because we used the mean of strain values we are able to calculate strain/genotype means, which reduce the ‘noise’ from strain effects and allow us to map QTL. This is reflected in the $H_{\mathrm{RI}\bar{x}}^{2}$ where the number of strains is used to reduce the impact of environmental and stochastic developmental sources of variance (Belknap, 1998).

In our analysis of the BXD population, parent-of-origin effects, particularly maternal effects, are lost within this overarching litter effect. It is only in the diallele cross, and looking at the reciprocal F1 females, that we are able to identify definitive parent-of-origin effects, and even here we are not able to narrow these down further to specific types of parent-of-origin effects. To truly tease apart pre-natal IGEs, postnatal IGEs, imprinting, and other postnatal litter effects a large multicomponent study would be required. In this study, mothers of every strain would have eggs (or embryos) transferred from mothers of every other strain (allowing prenatal parent-of-origin to be identified), and then each of these mother–offspring strain combinations would have to be cross-fostered postnatally to every maternal strain (to allow identification of postnatal parent-of-origin effects, and the epistatic interactions between pre- and post-natal effects). Something similar to this has been done on a much smaller scale by Cowley et al. (1989), although they did not investigate a sufficient numbers of strains to be able to do a genetic analysis. To tease apart strain effects and maternal effects, a home-cage monitoring system would be required to monitor the behavior of all pups, their interactions with each other, and their interactions with the foster mother. Again, perhaps a machine learning approach would allow a feasible analysis of this much home cage data, and to co-analyze this with USV data.

We did not see a correlation between our USV data and maternal care data previously collected in the BXD family (Ashbrook et al., 2015a, 2017). Although this may be due to simple logistical differences, i.e., only using ∼18 strains in common between the two experiments, it could also be due to biological reasons. As mentioned above, it is well recorded that the D2 strain have hearing loss at a relatively early age (Cohen-Salmon et al., 1985; Zheng et al., 1999), and therefore approximately half of the BXD population would be expected to have this early age hearing loss. This may result in co-adaptation, such that strains which have hearing loss use other, non-vocal, methods to solicit maternal provisioning. On-going work is investigating hearing in the entire BXD population, and future studies should combine analysis of infant USVs, maternal hearing, and mother–offspring interactions to provide a full picture.

## Conclusion

We have produced heritability estimates for several features of infant vocalization in mice, and defined QTLs underlying some of these core innate behaviors. All contain plausible candidate genes (Supplementary Material), but the ultimate aim of this strategy is to systematically move from QTLs, to genes, to mechanisms, and, potentially, translation to humans. This is obviously a long and complex process, but here we have taken the first step in defining phenotypes and identifying linked genomic regions. These regions will need to be confirmed with other methods, such as complimentary mouse populations, or, the potentially more useful method, reverse genetics, such as using CRISPR to insert the *B* or *D* allele in otherwise genetically identical strains. If large GWASs of infant vocalizations are carried out in humans, then they would provide an excellent dataset for comparison with this work, as we have done for other traits (Ashbrook et al., 2014, 2015b; Williams and Auwerx, 2015; Huang et al., 2018, unpublished).

Understanding infant vocalizations in mice is not just important for understanding the evolution of mother–offspring interactions, but also in understanding vocalization in human infants, perhaps with applications to the understanding and early identification of developmental disorders, such as autism-spectrum disorders.

## Author Contributions

TR, MS, DH, LL, and RW contributed to the conception and design of the study. SR, BC, and TR collected the data. BC, TR, and DA performed the statistical analyses. DA and SR wrote the first draft of the manuscript. SR, BC, TR, DA, and RW wrote sections of the manuscript. MS and TR contributed to the interpretation of the data. All authors contributed to manuscript revision, read and approved the submitted version.

## Conflict of Interest Statement

The authors declare that the research was conducted in the absence of any commercial or financial relationships that could be construed as a potential conflict of interest.

## Footnotes

1 http://translatingtime.org/translate

2 www.avisoft.com

3 www.braintreesci.com

4 www.genenetwork.org/webqtl/main.py?FormID=sharinginfo&GN_AccessionId=600

5 gn2.genenetwork.org

6 github.com/nickFurlotte/pylmm

7 http://kbroman.org/qtl2/

8 www.informatics.jax.org/allele

9 www.ncbi.nlm.nih.gov/gene

10 www.translatingtime.org

## References

[B1] Abramowitz L. K. Bartolomei M. S. (2012). Genomic imprinting: recognition and marking of imprinted loci. *Curr. Opin. Genet. Dev.* 22 72–78. 10.1016/j.gde.2011.12.001 22195775 PMC3314145

[B2] Alberts R. Schughart K. (2010). QTLminer: identifying genes regulating quantitative traits. *BMC Bioinformatics* 11:516. 10.1186/1471-2105-11-516 20950438 PMC2964687

[B3] Allen Institute for Brain Science (2008). *Allen Developing Mouse Brain Atlas.* Available at: http://developingmouse.brain-map.org/ [accessed January 7 2018]

[B4] Arriaga G. (2014). “Why the caged mouse sings: studies of the mouse ultrasonic song system and vocal behavior,” in *Biocommunication of Animals* ed. Witzany G. (Dordrecht: Springer) 81–101. 10.1007/978-94-007-7414-8_6

[B5] Ashbrook D. G. Gini B. Hager R. (2015a). Genetic variation in offspring indirectly influences the quality of maternal behaviour in mice. *Elife* 4:e11814. 10.7554/eLife.11814 26701914 PMC4758942

[B6] Ashbrook D. G. Hager R. (2013). Empirical testing of hypotheses about the evolution of genomic imprinting in mammals. *Front. Neuroanat.* 7:6. 10.3389/fnana.2013.00006 23641202 PMC3639422

[B7] Ashbrook D. G. Hager R. (2017). “Social interactions and indirect genetic effects on complex juvenile and adult traits,” in *Methods in Molecular Biology (Clifton, N.J.)* eds Schughart K. Williams R. W. (New York, NY: Springer) 499–517. 10.1007/978-1-4939-6427-7_24 27933541

[B8] Ashbrook D. G. Sharmin N. Hager R. (2017). Offspring genes indirectly influence sibling and maternal beha vioural strategies over resource share. *Proc. R. Soc. B Biol. Sci.* 284:20171059. 10.1098/rspb.2017.1059 28954905 PMC5627198

[B9] Ashbrook D. G. Williams R. W. Lu L. Hager R. (2015b). A cross-species genetic analysis identifies candidate genes for mouse anxiety and human bipolar disorder. *Front. Behav. Neurosci.* 9:171. 10.3389/fnbeh.2015.00171 26190982 PMC4486840

[B10] Ashbrook D. G. Williams R. W. Lu L. Stein J. L. Hibar D. P. Nichols T. E. (2014). Joint genetic analysis of hippocampal size in mouse and human identifies a novel gene linked to neurodegenerative disease. *BMC Genomics* 15:850. 10.1186/1471-2164-15-850 25280473 PMC4192369

[B11] Ashburner M. Ball C. A. Blake J. A. Botstein D. Butler H. Cherry J. M. (2000). Gene ontology: tool for the unification of biology. The Gene Ontology Consortium. *Nat. Genet.* 25 25–29. 10.1038/75556 10802651 PMC3037419

[B12] Barlow D. P. (2011). Genomic imprinting: a mammalian epigenetic discovery model. *Annu. Rev. Genet.* 45 379–403. 10.1146/annurev-genet-110410-132459 21942369

[B13] Baud A. Mulligan M. K. Casale F. P. Ingels J. F. Bohl C. J. Callebert J. (2017). Genetic variation in the social environment contributes to health and disease. *PLoS Genet.* 13:e1006498. 10.1371/journal.pgen.1006498 28121987 PMC5266220

[B14] Belknap J. K. (1998). Effect of within-strain sample size on QTL detection and mapping using recombinant inbred mouse strains. *Behav. Genet.* 28 29–38. 10.1023/A:1021404714631 9573644

[B15] Belknap J. K. Mitchell S. R. O’Toole L. A. Helms M. L. Crabbe J. C. (1996). Type I and type II error rates for quantitative trait loci (QTL) mapping studies using recombinant inbred mouse strains. *Behav. Genet.* 26 149–160. 10.1007/BF02359892 8639150

[B16] Bell R. W. Nitschke W. Zachman T. A. (1972). Ultra-sounds in three inbred strains of young mice. *Behav. Biol.* 7 805–814. 10.1016/S0091-6773(72)80172-X 4655399

[B17] Bello S. M. Smith C. L. Eppig J. T. (2015). Allele, phenotype and disease data at mouse genome informatics: improving access and analysis. *Mamm. Genome* 26 285–294. 10.1007/s00335-015-9582-y 26162703 PMC4534497

[B18] Blumstein D. T. Nguyen K. T. Martin J. G. A. (2013). Ontogenetic variation of heritability and maternal effects in yellow-bellied marmot alarm calls. *Proc. Biol. Sci.* 280:20130176. 10.1098/rspb.2013.0176 23466987 PMC3619468

[B19] Branchi I. Santucci D. Alleva E. (2001). Ultrasonic vocalisation emitted by infant rodents: a tool for assessment of neurobehavioural development. *Behav. Brain Res.* 125 49–56. 10.1016/S0166-4328(01)00277-7 11682093

[B20] Brunelli S. A. Curley J. P. Gudsnuk K. Champagne F. A. Myers M. M. Hofer M. A. (2015). Variations in maternal behavior in rats selected for infant ultrasonic vocalization in isolation. *Horm. Behav.* 75 78–83. 10.1016/j.yhbeh.2015.08.007 26306860

[B21] Carneiro A. M. D. Airey D. C. Thompson B. Zhu C.-B. Lu L. Chesler E. J. (2009). Functional coding variation in recombinant inbred mouse lines reveals multiple serotonin transporter-associated phenotypes. *Proc. Natl. Acad. Sci. U.S.A.* 106 2047–2052. 10.1073/pnas.0809449106 19179283 PMC2632716

[B22] Chai S. Gillombardo C. B. Donovan L. Strohl K. P. (2011). Morphological differences of the carotid body among C57/BL6 (B6), A/J, and CSS B6A1 mouse strains. *Respir. Physiol. Neurobiol.* 177 265–272. 10.1016/j.resp.2011.04.021 21555000 PMC4455900

[B23] Chittora A. Patil H. A. (2017). Data collection of infant cries for research and analysis. *J. Voice* 31 252.e15–252.e26. 10.1016/j.jvoice.2016.07.007 27658339

[B24] Cohen-Salmon C. Carlier M. Roubertoux P. Jouhaneau J. Semal C. Paillette M. (1985). Differences in patterns of pup care in mice. V–Pup ultrasonic emissions and pup care behavior. *Physiol. Behav.* 35 167–174. 10.1016/0031-9384(85)90331-2 4070379

[B25] Cowley D. E. Pomp D. Atchley W. R. Eisen E. J. Hawkins-Brown D. (1989). The impact of maternal uterine genotype on postnatal growth and adult body size in mice. *Genetics* 122 193–203. 2731729 10.1093/genetics/122.1.193 PMC1203684

[B26] Curry T. Egeto P. Wang H. Podnos A. Wasserman D. Yeomans J. (2013). Dopamine receptor D2 deficiency reduces mouse pup ultrasonic vocalizations and maternal responsiveness. *Genes Brain. Behav.* 12 397–404. 10.1111/gbb.12037 23521753

[B27] D’Amato F. R. Populin R. (1987). Mother-offspring interaction and pup development in genetically deaf mice. *Behav. Genet.* 17 465–475. 10.1007/BF01073113 3426503

[B28] Delprato A. Algéo M.-P. Bonheur B. Bubier J. A. Lu L. Williams R. W. (2017). QTL and systems genetics analysis of mouse grooming and behavioral responses to novelty in an open field. *Genes. Brain. Behav.* 16 790–799. 10.1111/gbb.12392 28544613 PMC5800503

[B29] Doty R. L. (1974). A cry for the liberation of the female rodent: courtship and copulation in rodentia. *Psychol. Bull.* 81 159–172. 10.1037/h0035971 4594961

[B30] Ehret G. (2005). Infant rodent ultrasounds – a gate to the understanding of sound communication. *Behav. Genet.* 35 19–29. 10.1007/s10519-004-0853-8 15674530

[B31] Ehret G. Haack B. (1982). Ultrasound recognition in house mice: Key-Stimulus configuration and recognition mechanism. *J. Comp. Physiol.* 148 245–251. 10.1007/BF00619131

[B32] Esposito G. Hiroi N. Scattoni M. L. (2017). Cry, baby, cry: expression of distress as a biomarker and modulator in Autism spectrum disorder. *Int. J. Neuropsychopharmacol.* 20 498–503. 10.1093/ijnp/pyx014 28204487 PMC5458334

[B33] Forstmeier W. Burger C. Temnow K. Derégnaucourt S. (2009). The genetic basis of zebra finch vocalizations. *Evolution* 63 2114–2130. 10.1111/j.1558-5646.2009.00688.x 19453380

[B34] Fujita E. Tanabe Y. Shiota A. Ueda M. Suwa K. Momoi M. Y. (2008). Ultrasonic vocalization impairment of Foxp2 (R552H) knockin mice related to speech-language disorder and abnormality of Purkinje cells. *Proc. Natl. Acad. Sci. U.S.A.* 105 3117–3122. 10.1073/pnas.0712298105 18287060 PMC2268594

[B35] Gelfand A. A. (2016). Infant colic. *Semin. Pediatr. Neurol.* 23 79–82. 10.1016/j.spen.2015.08.003 27017027 PMC4809021

[B36] Gene Ontology Consortium. (2015). Gene ontology consortium: going forward. *Nucleic Acids Res.* 43 D1049–D1056. 10.1093/nar/gku1179 25428369 PMC4383973

[B37] Gustafsson L. (1986). Lifetime reproductive success and heritability: empirical support for fisher’s fundamental theorem. *Am. Nat.* 128 761–764. 10.1086/284601

[B38] Hahn M. E. Hewitt J. K. Adams M. Tully T. (1987). Genetic influences on ultrasonic vocalizations in young mice. *Behav. Genet.* 17 155–166. 10.1007/BF01065994 3606538

[B39] Hahn M. E. Hewitt J. K. Schanz N. Weinreb L. Henry A. (1997). Genetic and developmental influences on infant mouse ultrasonic calling. I. A diallel analysis of the calls of 3-day olds. *Behav. Genet.* 27 133–143. 10.1023/A:1025637408900 9145552

[B40] Haley C. S. Knott S. A. (1992). A simple regression method for mapping quantitative trait loci in line crosses using flanking markers. *Heredity* 69 315–324. 10.1038/hdy.1992.131 16718932

[B41] Hegoburu C. Shionoya K. Garcia S. Messaoudi B. Thévenet M. Mouly A.-M. (2011). The RUB cage: respiration-ultrasonic vocalizations-behavior acquisition setup for assessing emotional memory in rats. *Front. Behav. Neurosci.* 5:25. 10.3389/fnbeh.2011.00025 21637320 PMC3101376

[B42] Heiderstadt K. M. Vandenbergh D. J. Gyekis J. P. Blizard D. A. (2014). Communal nesting increases pup growth but has limited effects on adult behavior and neurophysiology in inbred mice. *J. Am. Assoc. Lab. Anim. Sci.* 53 152–160. 24602541 PMC3966271

[B43] Hennessy M. B. Li J. Lowe E. L. Levine S. (1980). Maternal behavior, pup vocalizations, and pup temperature changes following handling in mice of 2 inbred strains. *Dev. Psychobiol.* 13 573–584. 10.1002/dev.420130603 7429018

[B44] Hernandez-Miranda L. R. Ruffault P.-L. Bouvier J. C. Murray A. J. Morin-Surun M.-P. Zampieri N. (2017). Genetic identification of a hindbrain nucleus essential for innate vocalization. *Proc. Natl. Acad. Sci. U.S.A.* 114 8095–8100. 10.1073/pnas.1702893114 28698373 PMC5544295

[B45] Insel T. R. Hill J. L. Mayor R. B. (1986). Rat pup ultrasonic isolation calls: possible mediation by the benzodiazepine receptor complex. *Pharmacol. Biochem. Behav.* 24 1263–1267. 10.1016/0091-3057(86)90182-6 2425378

[B46] Kanehisa M. Goto S. (2000). KEGG: kyoto encyclopedia of genes and genomes. *Nucleic Acids Res.* 28 27–30. 10.1093/nar/28.1.27 10592173 PMC102409

[B47] Kanehisa M. Goto S. Sato Y. Furumichi M. Tanabe M. (2012). KEGG for integration and interpretation of large-scale molecular data sets. *Nucleic Acids Res.* 40 D109–D114. 10.1093/nar/gkr988 22080510 PMC3245020

[B48] Knoll A. T. Jiang K. Levitt P. (2017). QTL mapping and analysis of heritable variation in affiliative social behavior and related traits. *Genes Brain Behav.* 17:e12431. 10.1111/gbb.12431 29052939 PMC5910301

[B49] Knoll A. T. Jiang K. Levitt P. (2018). Quantitative trait locus mapping and analysis of heritable variation in affiliative social behavior and co-occurring traits. *Genes. Brain. Behav.* 17:e12431. 10.1111/gbb.12431 29052939 PMC5910301

[B50] Lingle S. Riede T. (2014). Deer mothers are sensitive to infant distress vocalizations of diverse mammalian species. *Am. Nat.* 184 510–522. 10.1086/677677 25226186

[B51] Lingle S. Wyman M. T. Kotrba R. Teichroeb L. J. Romanow C. A. (2012). What makes a cry a cry? A review of infant distress vocalizations. *Curr. Zool.* 58 698–726. 10.1093/czoolo/58.5.698

[B52] Maggio J. C. Whitney G. (1986). Heterosis of adult mouse (Mus musculus) ultrasonic vocalizing. *Behav. Genet.* 16 493–506. 10.1007/BF01074267 3753377

[B53] Manichaikul A. Dupuis J. Sen S. Broman K. W. (2006). Poor performance of bootstrap confidence intervals for the location of a quantitative trait locus. *Genetics* 174 481–489. 10.1534/genetics.106.061549 16783000 PMC1569776

[B54] McCracken G. F. Gustin M. K. (2010). Nursing behavior in mexican free-tailed bat maternity colonies. *Ethology* 89 305–321. 10.1111/j.1439-0310.1991.tb00376.x

[B55] Mogi K. Takakuda A. Tsukamoto C. Ooyama R. Okabe S. Koshida N. (2017). Mutual mother-infant recognition in mice: the role of pup ultrasonic vocalizations. *Behav. Brain Res.* 325 138–146. 10.1016/j.bbr.2016.08.044 27567527

[B56] Moore A. J. Brodie E. D. III Wolf J. B. (1997). Interacting phenotypes and the evolutionary process: I. Direct and indirect genetic effects of social interactions. *Evolution* 51 1352–1362. 10.2307/2411187 28568644

[B57] Mozhui K. Karlsson R.-M. Kash T. L. Ihne J. Norcross M. Patel S. (2010). Strain differences in stress responsivity are associated with divergent amygdala gene expression and glutamate-mediated neuronal excitability. *J. Neurosci.* 30 5357–5367. 10.1523/JNEUROSCI.5017-09.2010 20392957 PMC2866495

[B58] Nakagawa R. Matsunaga E. Okanoya K. (2012). Defects in ultrasonic vocalization of cadherin-6 knockout mice. *PLoS One* 7:e49233. 10.1371/journal.pone.0049233 23173049 PMC3500271

[B59] Noirot E. (1964). Changes in responsiveness to young in the adult mouse: The effect of external stimuli. *J. Comp. Physiol. Psychol.* 57 97–99. 10.1037/h0042864 14125097

[B60] Noirot E. (1966). Ultra-sounds in young rodents. I. Changes with age in albino mice. *Anim. Behav.* 14 459–462. 10.1016/S0003-3472(66)80045-3 5972804

[B61] Okon E. E. (1970a). The effect of environmental temperature on the production of ultrasounds by isolated non-handled albino mouse pups. *J. Zool.* 162 71–83. 10.1111/j.1469-7998.1970.tb01258.x

[B62] Okon E. E. (1970b). The ultrasonic responses of albino mouse pups to tactile stimuli. *J. Zool.* 162 485–492. 10.1111/j.1469-7998.1970.tb01283.x

[B63] Oller D. K. Buder E. H. Ramsdell H. L. Warlaumont A. S. Chorna L. Bakeman R. (2013). Functional flexibility of infant vocalization and the emergence of language. *Proc. Natl. Acad. Sci. U.S.A.* 110 6318–6323. 10.1073/pnas.1300337110 23550164 PMC3631625

[B64] Peirce J. L. Lu L. Gu J. Silver L. M. Williams R. W. (2004). A new set of BXD recombinant inbred lines from advanced intercross populations in mice. *BMC Genet.* 5:7. 10.1186/1471-2156-5-7 15117419 PMC420238

[B65] Philip V. M. Duvvuru S. Gomero B. Ansah T. A. Blaha C. D. Cook M. N. (2010). High-throughput behavioral phenotyping in the expanded panel of BXD recombinant inbred strains. *Genes Brain. Behav.* 9 129–159. 10.1111/j.1601-183X.2009.00540.x 19958391 PMC2855868

[B66] Price T. Schluter D. (1991). On the low heritability of life-history traits. *Evolution* 45 853–861. 10.1111/j.1558-5646.1991.tb04354.x 28564058

[B67] Riede T. (2011). Subglottal pressure, tracheal airflow, and intrinsic laryngeal muscle activity during rat ultrasound vocalization. *J. Neurophysiol.* 106 2580–2592. 10.1152/jn.00478.2011 21832032 PMC3214115

[B68] Riede T. (2013). Stereotypic laryngeal and respiratory motor patterns generate different call types in rat ultrasound vocalization. *J. Exp. Zool. A Ecol. Genet. Physiol.* 319 213–224. 10.1002/jez.1785 23423862 PMC3926509

[B69] Riede T. Borgard H. L. Pasch B. (2017). Laryngeal airway reconstruction indicates that rodent ultrasonic vocalizations are produced by an edge-tone mechanism. *R. Soc. Open Sci.* 4:170976. 10.1098/rsos.170976 29291091 PMC5717665

[B70] Riede T. Brown C. (2013). Body size, vocal fold length, and fundamental frequency – implications for mammal vocal communication. *Nova. Acta Leopold.* 380 295–314.

[B71] Riede T. Zhao Y. LeDoux M. S. (2015). Vocal development in dystonic rats. *Physiol. Rep.* 3:e12350. 10.14814/phy2.12350 25907786 PMC4425958

[B72] Roberts L. H. (1975). Evidence for the laryngeal source of ultrasonic and audible cries of rodents. *J. Zool.* 175 243–257. 10.1111/j.1469-7998.1975.tb01399.x

[B73] Rosen G. D. Pung C. J. Owens C. B. Caplow J. Kim H. Mozhui K. (2009). Genetic modulation of striatal volume by loci on Chrs 6 and 17 in BXD recombinant inbred mice. *Genes Brain Behav.* 8 296–308. 10.1111/j.1601-183X.2009.00473.x 19191878 PMC2706028

[B74] Roubertoux P. L. Martin B. Le Roy I. Beau J. Marchaland C. Perez-Diaz F. (1996). Vocalizations in newborn mice: genetic analysis. *Behav. Genet.* 26 427–437. 10.1007/BF02359487 8771903

[B75] Roy S. Watkins N. Heck D. (2012). Comprehensive analysis of ultrasonic vocalizations in a mouse model of fragile X syndrome reveals limited, call type specific deficits. *PLoS One* 7:e44816. 10.1371/journal.pone.0044816 22984567 PMC3439444

[B76] Scattoni M. L. Crawley J. Ricceri L. (2009). Ultrasonic vocalizations: a tool for behavioural phenotyping of mouse models of neurodevelopmental disorders. *Neurosci. Biobehav. Rev.* 33 508–515. 10.1016/j.neubiorev.2008.08.003 18771687 PMC2688771

[B77] Scattoni M. L. Gandhy S. U. Ricceri L. Crawley J. N. (2008a). Unusual repertoire of vocalizations in the BTBR T + tf/J mouse model of autism. *PLoS One* 3:e3067. 10.1371/journal.pone.0003067 18728777 PMC2516927

[B78] Scattoni M. L. McFarlane H. G. Zhodzishsky V. Caldwell H. K. Young W. S. Ricceri L. (2008b). Reduced ultrasonic vocalizations in vasopressin 1b knockout mice. *Behav. Brain Res.* 187 371–378. 10.1016/j.bbr.2007.09.034 18005969 PMC2255061

[B79] Sheinkopf S. J. Iverson J. M. Rinaldi M. L. Lester B. M. (2012). Atypical cry acoustics in 6-month-old infants at risk for autism spectrum disorder. *Autism Res.* 5 331–339. 10.1002/aur.1244 22890558 PMC3517274

[B80] Shete S. Beasley T. M. Etzel C. J. Fernández J. R. Chen J. Allison D. B. (2004). Effect of winsorization on power and type 1 error of variance components and related methods of QTL detection. *Behav. Genet.* 34 153–159. 10.1023/B:BEGE.0000013729.26354.da 14755180

[B81] Shu W. Cho J. Y. Jiang Y. Zhang M. Weisz D. Elder G. A. (2005). Altered ultrasonic vocalization in mice with a disruption in the Foxp2 gene. *Proc. Natl. Acad. Sci. U.S.A.* 102 9643–9648. 10.1073/pnas.0503739102 15983371 PMC1160518

[B82] Simecek P. Forejt J. Williams R. W. Shiroishi T. Takada T. Lu L. (2017). High-resolution maps of mouse reference populations. *G*3 7 3427–3434. 10.1534/g3.117.300188 28839117 PMC5633391

[B83] Sirotin Y. B. Costa M. E. Laplagne D. A. (2014). Rodent ultrasonic vocalizations are bound to active sniffing behavior. *Front. Behav. Neurosci.* 8:399. 10.3389/fnbeh.2014.00399 25477796 PMC4235378

[B84] Sloan Z. Arends D. W. Broman K. Centeno A. Furlotte N. Nijveen H. (2016). GeneNetwork: framework for web-based genetics. *J. Open Source Softw.* 1:25 10.21105/joss.00025

[B85] Smarius L. J. C. A. Strieder T. G. A. Loomans E. M. Doreleijers T. A. H. Vrijkotte T. G. M. Gemke R. J. (2017). Excessive infant crying doubles the risk of mood and behavioral problems at age 5: evidence for mediation by maternal characteristics. *Eur. Child Adolesc. Psychiatry* 26 293–302. 10.1007/s00787-016-0888-4 27422707 PMC5323467

[B86] Smith J. C. (1976). Responses of adult mice to models of infant calls. *J. Comp. Physiol. Psychol.* 90 1105–1115. 10.1037/h0077287 20375884

[B87] Spence H. R. Aslam A. M. Hofer M. A. Brunelli S. A. Shair H. N. (2016). Vocal coselection in rat pup ultrasonic vocalizations. *Ecol. Evol.* 6 1922–1929. 10.1002/ece3.1907 27066218 PMC4767333

[B88] Sul J. H. Bilow M. Yang W.-Y. Kostem E. Furlotte N. He D. (2016). Accounting for population structure in gene-by-environment interactions in genome-wide association studies using mixed model. *PLoS Genet.* 12:e1005849. 10.1371/journal.pgen.1005849 26943367 PMC4778803

[B89] Sunkin S. M. Ng L. Lau C. Dolbeare T. Gilbert T. L. Thompson C. L. (2013). Allen Brain Atlas: an integrated spatio-temporal portal for exploring the central nervous system. *Nucleic Acids Res.* 41 D996–D1008. 10.1093/nar/gks1042 23193282 PMC3531093

[B90] Thornton L. M. Hahn M. E. Schanz N. (2005). Genetic and developmental influences on infant mouse ultrasonic calling. III. Patterns of inheritance in the calls of mice 3-9 days of age. *Behav. Genet.* 35 73–83. 10.1007/s10519-004-0857-4 15674534

[B91] Van Segbroeck M. Knoll A. T. Levitt P. Narayanan S. (2017). MUPET—mouse ultrasonic profile extraction: a signal processing tool for rapid and unsupervised analysis of ultrasonic vocalizations. *Neuron* 94 465–485. 10.1016/j.neuron.2017.04.005 28472651 PMC5939957

[B92] Vias C. Dick A. S. (2017). Cerebellar contributions to language in typical and atypical development: a review. *Dev. Neuropsychol.* 42 404–421. 10.1080/87565641.2017.1334783 28885046 PMC6232854

[B93] Walsh B. Lynch M. (2018). *Evolution and Selection of Quantitative Traits.* New York, NY: Oxford University Press 132–133 333–334 10.1093/oso/9780198830870.001.0001

[B94] Wang H. Liang S. Burgdorf J. Wess J. Yeomans J. (2008). Ultrasonic vocalizations induced by sex and amphetamine in M2, M4, M5 muscarinic and D2 dopamine receptor knockout mice. *PLoS One* 3:e1893. 10.1371/journal.pone.0001893 18382674 PMC2268741

[B95] Wang X. Pandey A. K. Mulligan M. K. Williams E. G. Mozhui K. Li Z. (2016). Joint mouse-human phenome-wide association to test gene function and disease risk. *Nat. Commun.* 7:10464. 10.1038/ncomms10464 26833085 PMC4740880

[B96] Weidt A. Lindholm A. K. König B. (2014). Communal nursing in wild house mice is not a by-product of group living: females choose. *Naturwissenschaften* 101 73–76. 10.1007/s00114-013-1130-6 24389536 PMC3893474

[B97] Wickham H. (2016). *ggplot2: Elegant Graphics for Data Analysis.* New York, NY: Springer-Verlag 10.1007/978-3-319-24277-4

[B98] Williams E. G. Auwerx J. (2015). The convergence of systems and reductionist approaches in complex trait analysis. *Cell* 162 23–32. 10.1016/j.cell.2015.06.024 26140590 PMC4493761

[B99] Wöhr M. (2014). Ultrasonic vocalizations in Shank mouse models for autism spectrum disorders: detailed spectrographic analyses and developmental profiles. *Neurosci. Biobehav. Rev.* 43 199–212. 10.1016/j.neubiorev.2014.03.021 24726578

[B100] Wöhr M. Dahlhoff M. Wolf E. Holsboer F. Schwarting R. K. W. Wotjak C. T. (2008). Effects of genetic background, gender, and early environmental factors on isolation-induced ultrasonic calling in mouse pups: an embryo-transfer study. *Behav. Genet.* 38 579–595. 10.1007/s10519-008-9221-4 18712592

[B101] Workman A. D. Charvet C. J. Clancy B. Darlington R. B. Finlay B. L. (2013). Modeling transformations of neurodevelopmental sequences across mammalian species. *J. Neurosci.* 33 7368–7383. 10.1523/JNEUROSCI.5746-12.2013 23616543 PMC3928428

[B102] Xue Y. Li J. Yan L. Lu L. Liao F.-F. (2015). Genetic variability to diet-induced hippocampal dysfunction in BXD recombinant inbred (RI) mouse strains. *Behav. Brain Res.* 292 83–94. 10.1016/j.bbr.2015.06.023 26092713 PMC5226443

[B103] Yamauchi M. Ocak H. Dostal J. Jacono F. J. Loparo K. A. Strohl K. P. (2008). Post-sigh breathing behavior and spontaneous pauses in the C57BL/6J (B6) mouse. *Respir. Physiol. Neurobiol.* 162 117–125. 10.1016/j.resp.2008.05.003 18565803 PMC3698969

[B104] Zheng Q. Y. Johnson K. R. Erway L. C. (1999). Assessment of hearing in 80 inbred strains of mice by ABR threshold analyses. *Hear. Res.* 130 94–107. 10.1016/S0378-5955(99)00003-9 10320101 PMC2855304

[B105] Zhou X. Stephens M. (2012). Genome-wide efficient mixed-model analysis for association studies. *Nat. Genet.* 44 821–824. 10.1038/ng.2310 22706312 PMC3386377

