# Supplementary material for: Born to Cry: A Genetic Dissection of Infant Vocalization
Source: Front Behav Neurosci. 2018 Oct 29;12:250. doi: 10.3389/fnbeh.2018.00250 (PMC6216097; doi:10.3389/fnbeh.2018.00250)
Supplement: Supplementary file 5 [file Data_Sheet_1.docx]

Supplementary Material

**Born to cry: A genetic dissection of infant vocalization**

David George Ashbrook^*^, Snigdha Roy, Brittany G. Clifford, Tobias Riede, Maria Luisa Scattoni, Detlef H. Heck, Lu Lu, Robert W. Williams

*Correspondence:

David G. Ashbrook

dashbrook@UTHSC.edu

**Supplementary methods**

**Correlations with maternal care and pup solicitation behavior**

Infant USVs are known to increase maternal care (Noirot 1964; Smith 1976; Ehret & Haack 1982; Cohen-Salmon *et al.* 1985). Indirect genetic effects on maternal care, and direct genetic effects on pup solicitation behavior have been previously measured in a subset of BXD strains in a different cohort (Ashbrook *et al.* 2015, 2017) and are available in GeneNetwork. To identify if vocalizations correlated with solicitation, we computed correlations between traits (solicitation traits: GN IDs 18789, 18781, 18785, 18787, 18786, 18541, 18539, 18535, 18532) and our new data (vocalization traits: 16359, 16360, 16600, 16602, 16357, 16358, 16362, 16603, 16355, 16356, 16361, 16610, 16611, 16612, 16617).

**Supplementary results**

**Correlations with maternal care and pup solicitation behavior**

Indirect genetic effects on maternal care, and direct genetic effects on pup solicitation behavior have been previously measured in a separate study using a subset of BXD strains which overlap with those we have used to measure USVs (Ashbrook *et al.* 2015, 2017). Therefore, we performed correlations between these traits and vocalization traits to identify if USVs correlated with solicitation (e.g. do higher vocalization rates correlate with increased behavioral measures of solicitation), or if pups that vocalize more receive more maternal care. There was only one nominally significant correlation: an indirect genetic effect of BXD foster-offspring genotype on the amount of suckling the mother provided to B6 foster offspring on postnatal day 14 (GN 18787) and mean peak frequency of call on day 9 (GN 16600; *n* = 19, ρ = –0.619, *p* = 3.80E-03). However, this relationship was not significant when corrected for the number of comparisons being made.

**Supplementary discussion**

**Correlations with maternal care and pup solicitation behavior**

The lack of correlations between the vocalization traits we measured and previous maternal care traits could have several reasons. Firstly, the overlap between strains used in both experiments was low (n = 18-19), and this reduces our power to detect correlations, as even small errors in measurement will have a large effect. Secondly, previous work has shown that maternal care can be initiated by other cues, not just USVs (D’Amato & Populin 1987). In relation to this, within each strain there will have been selective pressure during inbreeding for pups from strains with low USVs to be selected to increase other signals (e.g. odor, touch), or mothers from strains with low USVs to be selected to be more sensitive to these other signals. One of the parental strains to the BXD, D2, has been found to impaired hearing at the frequencies of pup USVs (Cohen-Salmon *et al.* 1985), and therefore it would be expected that some of the BXD strains will also impaired hearing at these frequencies. As there is a correlation between deafness and lower pup USVs (D’Amato & Populin 1987), it would be interesting to examine if BXD strains with low USVs have impaired hearing, and as such, it would be an important follow-up to test the hearing range of BXD strains. However, we see no evidence that the D2 mice used here were completely deaf: D2 pups vocalized more than their B6 equivalents, and D2 mothers were equally able to respond to offspring as B6 mothers. If the D2 mice do have hearing impairment, then this might explain the difference in vocalizations we see here: the D2 offspring may have been selected to produce more, louder calls of longer duration at a lower frequency, as these may be easier for mothers with partial hearing impairments to hear. This is not important in the current study, as we are simply interested in the genetic determinants of USVs, not the responses they cause.

**Candidate gene selection**

We identified three significant QTL, and below we examine some of the genes found within those QTL which have links to our phenotypes within the literature.

The first of our QTL, on Chr 14, contained too many genes to provide good candidates. However, there is a human locus linked to ‘non-word repetition’ (a language test which can be applied across ages) on Chr 13 (Truong *et al.* 2016) from ~52 and 55 cM (equivalent to ~48 – 53.5 Mb). This locus contains genes found in four homologous mouse regions: Chr 8 21.9 – 22.3 Mbp, Chr 14:59.2 – 63, Chr 14:72.5 – 73.6 and Chr 14:79.6 – 79.8 Mb. As we can see, these Chr 14 intervals overlap with our Chr 14 QTL ~40 – 73 Mb: further, the two intervals from ~59-63 and 72.5 – 73.5 Mb actually match the two peaks we see in this QTL. This human language QTL also overlaps with a previous locus found for specific language impairment (Bartlett *et al.* 2002, 2004), and autism (Bradford *et al.* 2001; Smith *et al.* 2002). If we accept this hypothesis that homologous genes influence mouse vocalization and human speech, fourteen of the genes within this human locus have a nsSNP or indel in the homologous mouse genes, nine in the 59 – 63 Mb region (*RCBTB1, CAB39L, EBPL, SPRYD7, DLEU2, GUCY1B2, FAM124A, SERPINE3, WDFY2*) and five in the 72.5 – 73.5 Mb region (*FNDC3A, CYSLTR2, RCBTB2, RB1, LPAR6*). Therefore, these fourteen genes are candidates underlying the QTL. We should note that none of these fourteen overlap the eight which the authors of the previous paper (Truong *et al.* 2016) highlighted as candidates due to known neurological or cognitive function.

Another gene potentially underlying our QTL is *Fgf17*, knockout of which has been reported to cause a reduction in vocalization (Scearce-Levie *et al.* 2008). *Fgf17* lies at ~ 70.6 Mb, and therefore within our QTL. However, there is no known *cis*-eQTLs, nsSNP or indel in *Fgf17* in the BXD. Deep sequencing of the BXD is ongoing: if any previously unknown variants are found in *Fgf17* it may become a better candidate. We should however, point out that Scearce-Levie et al. give very little description of how their knockout was produced and confirmed, and if the knockout was larger than expected (altering another gene in the region) or if loci from the donor strain were in linkage with the knockout (it was produced on a 129X1/SvJ background, and only crossed to C57BL/6NCrl for one generation), then any phenotypic changes may actually be due to other, nearby, genes. This give us confidence that this area may be involved in vocalization, but does not help us determine the causal variant.

Our second QTL, on Chr 2, has a much smaller confidence interval than the QTL above, containing only 44 genes, and therefore we can identify the genes within it as potential candidates, and highlight some of the stronger candidates (Supplementary table 1). A few of the genes within this QTL are of particular note.

*Ntsr1* (also referred to as *NT-1R*) has been linked to temperature homeostasis (Remaury *et al.* 2002), and it has been clearly shown that reduced temperature can increase infant USVs (Okon 1970). Further, *Ntsr1* knockout mice show alterations in anxiety-like behavior (Fitzpatrick *et al.* 2012), and agonists of the receptor cause anxiolytic-like effects on ultrasonic vocalizations in rats (Steele *et al.* 2017).

*Cdh4* is highly variable between the *B* and *D* alleles (35 nsSNPs and 57 indels) and knockout of another member of the cadherin gamily, *Cdh6*, has been shown to alter USVs (Nakagawa *et al.* 2012).

*Adrm1* (also known as *Rpn13*) knockout pups are less competitive for food compared to wildtype siblings (Al-Shami *et al.* 2010), and this could result in reduced ultrasonic vocalizations as the pups are less motivated to solicit the mother for food.

Finally, *Osbpl2* is mutated in autosomal hearing loss (Thoenes *et al.* 2015; Xing *et al.* 2015). Deaf mice, and mice raised by deaf mothers, produce fewer vocalizations than hearing mice (D’Amato & Populin 1987), and therefore this could indirectly cause the phenotype we detect.

Our third and final QTL was found on Chr 8, and influenced amplitude of USV. This QTL contains 185 genes, but only 27 of these have nsSNPs or indels, and so we concentrated on these as candidates.

*Csmd1* has been associated with several psychiatric disorders, and with abnormal emotion/affect behavior (Steen *et al.* 2013). As we mention above, anxiety-like behavior may be related to USVs. Further, *Csmd1* shows *cis*-eQTLs in several brain regions in BXD (striatum, neocortex, midbrain, hippocampus) and at different developmental points (striatum *cis*-eQTL at PN3, PN14 and adult). Additionally, there is a significant correlation between nucleus accumbens and midbrain expression of *Csmd1* and expression of the three phenotypes underlying this QTL (Supplementary table 2).

Copy number variants in *Dlgap2* has been associated with schizophrenia (Li *et al.* 2014) and autism (Pinto *et al.* 2010), and autism in turn has been linked to alterations in infant vocalization (Xu *et al.* 2009; Patten *et al.* 2014). Therefore, *Dlgap2* may be causing our change in vocalization: it would be interesting to see if other autism related traits, e.g. sociability, mapped to this locus in the BXD.

Finally, the gene *Cln8* may be of interest, as it has been linked to behavior (Bolivar *et al.* 2002), autism (Tylee *et al.* 2017) and food intake (Do *et al.* 2013), any of which could alter USVs.

**Supplementary figures**

Supplementary figure 1: Representative images of the ten different syllables examined in our qualitative analysis.

Supplementary figure 2: QTL maps produced using GeneNetwork for number of calls on postnatal days 7, 8, 9 and combined. The blue line represents the genome scan, showing the likelihood ratio statistic (LRS) associated with each marker across the locus. The top, pink, line marks genome-wide significance (genome-wide *p* ≤ 0.05), the lower, grey, line the suggestive significance threshold (genome-wide p ≤ 0.63). The green line show the additive coefficient, showing that the DBA/2J alleles increase trait values. The green axis on the right shows by how much the respective alleles increase trait values. Loci which were consistent between days were considered to be more reliable than those which were only seen on a single day.

Supplementary figure 3: QTL maps produced using GeneNetwork for duration of calls on postnatal days 7, 8, 9 and combined. The blue line represents the genome scan, showing the likelihood ratio statistic (LRS) associated with each marker across the locus. The top, pink, line marks genome-wide significance (genome-wide *p* ≤ 0.05), the lower, grey, line the suggestive significance threshold (genome-wide p ≤ 0.63). The green line show the additive coefficient, showing that the DBA/2J alleles increase trait values. The green axis on the right shows by how much the respective alleles increase trait values. Loci which were consistent between days were considered to be more reliable than those which were only seen on a single day.

Supplementary figure 4: QTL maps produced using GeneNetwork for frequency of calls on postnatal days 7, 8, 9 and combined. The blue line represents the genome scan, showing the likelihood ratio statistic (LRS) associated with each marker across the locus. The top, pink, line marks genome-wide significance (genome-wide *p* ≤ 0.05), the lower, grey, line the suggestive significance threshold (genome-wide p ≤ 0.63). The green line show the additive coefficient, showing that the DBA/2J alleles increase trait values. The green axis on the right shows by how much the respective alleles increase trait values. Loci which were consistent between days were considered to be more reliable than those which were only seen on a single day.

Supplementary figure 5: QTL maps produced using GeneNetwork for frequency of calls on postnatal days 7, 8, 9 and combined. The blue line represents the genome scan, showing the likelihood ratio statistic (LRS) associated with each marker across the locus. The top, pink, line marks genome-wide significance (genome-wide *p* ≤ 0.05), the lower, grey, line the suggestive significance threshold (genome-wide p ≤ 0.63). The green line show the additive coefficient, showing that the DBA/2J alleles increase trait values. The green axis on the right shows by how much the respective alleles increase trait values. Loci which were consistent between days were considered to be more reliable than those which were only seen on a single day.

Supplementary figure 6: Normal probability plots, a special case of the q-q plot, for all four quantitative vocalization traits in the BXD family, on postnatal days 7, 8 and 9. Plots were produced in GeneNetwork (genenetwork.org), and can be reproduced there.

Supplementary figure 7: Correlation between H^2^_RIx̅_ estimates calculated from the diallel cross and the BXD strains, showing a regression line blue with 95% confidence intervals (darker grey), and the Spearman’s rho.

Supplementary figure 8: Figure showing the power to detect a given effect size with a H^2^_RIx̅_ of 0.5.

Supplementary figure 9: Figure showing the power to detect a given effect size with a H^2^_RIx̅_ of 0.9

Supplementary figure 10: QTL map of the Chr 2 QTL for trait 16359, frequency of calls on postnatal day 7.

In the top section the location of genes are shown by yellow and purple blocks. Below this, the distribution of haplotype blocks in the 41 BXD strains is shown. Green represents the *D* allele, whereas red represents the *B* allele. It is clear that the majority of strains with high values have the *D* allele (green), whereas the majority of strains with the *B* allele (red) have low values.

In the bottom section, the blue line represents the genome scan, showing the likelihood ratio statistic (LRS) associated with each marker across the locus. The top, pink, line marks genome-wide significance (genome-wide *p* ≤ 0.05), the lower, grey, line the suggestive significance threshold (genome-wide p ≤ 0.63). The green line shows the additive coefficient, showing that the DBA/2J alleles increase trait values. The green axis on the right shows by how much the respective alleles increase trait values. The yellow bars represent the bootstap values, with the left edge of each bar showing where the peak fell in each of the 2000 resamples of the data.

Supplementary figure 11: QTL map of the Chr 8 QTL for trait 16357, amplitude of calls on postnatal day 7.

In the top section the location of genes are shown by yellow and purple blocks. Below this, the distribution of haplotype blocks in the 41 BXD strains is shown. Green represents the *D* allele, whereas red represents the *B* allele. It is clear that the majority of strains with high values have the *D* allele (green), whereas the majority of strains with the *B* allele (red) have low values.

In the bottom section, the blue line represents the genome scan, showing the likelihood ratio statistic (LRS) associated with each marker across the locus. The top, pink, line marks genome-wide significance (genome-wide *p* ≤ 0.05), the lower, grey, line the suggestive significance threshold (genome-wide p ≤ 0.63). The green line show the additive coefficient, showing that the DBA/2J alleles increase trait values. The green axis on the right shows by how much the respective alleles increase trait values. The yellow bars represent the bootstap values, with the left edge of each bar showing where the peak fell in each of the 2000 resamples of the data.

**Supplementary tables**

Supplementary table 1: Summary statistics of ANOVAs for strain, litter, sex, body weight and their interactions, for all four quantitative phenotypes (Number of calls, duration of calls, frequency of calls and peak amplitude of calls) measures in the diallel cross. The proportion and percentage of variance explained by each factor is also shown. The proportion of variance explained by strain is equivalent to *h^2^* whereas the proportion of variance explained by litter is the parental effect. Values are from 102 individuals, split into 6 litters of C57BL/6J  (B6) and DBA/2J (D2) strains, and 5 litters of each of the F1 genotypes.

Supplementary table 2: Summary statistics of ANOVAs for strain, litter, body weight and their interactions, for all twelve qualitative ultrasonic vocalization traits in the B6/D2 diallel cross, measured on postnatal day 8. These phenotypes were only measured on postnatal day 8. Values for 700 calls from B6, 1305 from D2, 2047 from B6D2F1 calls, and 1972 from D2B6F1. There were collected from 40 individuals, one male, one female, from five litters of each of the four genotypes. The proportion and percentage of variance explained by each factor is shown. The proportion of variance explained by strain is equivalent to *h^2^* whereas the proportion of variance explained by litter is the parental effect. For each phenotype, the significance of the Tukey *posthoc* test is shown for each pair of strains.

Supplementary table 3: Summary of candidate genes within the Chr 2 QTL. Gene names and their mm10 start locations are shown, along with phenotypes of interest from the Mouse Genome Informatics database and NCBI GeneRIFs and Pubmed abstracts. Presence of gene expression in the B6 postnatal day 4 brain from the Allen brain atlas developing brain map is shown (RSP = rostral secondary prosencephalon, tel = telencephalic vesicle, PHy = peduncular (caudal) hypothalamus, p3 = prosomere 3, p2 = prosomere 2, p1 = prosomere 1, M = midbrain, PPH = prepontine hindbrain, PH = pontine hindbrain, PMH = pontomedullary hindbrain, MH = medullary hindbrain). Gene expression data in the BXD family from GeneNetwork was used to identify brain regions with significant *cis-*eQTL and where there were correlations between gene expression and our measured traits of interest (brain regions used were postnatal day 3 neocortex, postnatal day 3 striatum, postnatal day 14 neocortex, postnatal day 14 striatum, adult amygdala, adult cerebellum, adult hippocampal precursor cells, adult hippocampus, adult hypothalamous, adult midbrain, adult neocortex, adult nucleus accumbens, adult prefrontal cortex, adult striatum, adult ventral tegmental area: PN = postnatal). Correlations were calculated within GeneNetwork using the Spearman correlation coefficient, ρ.

Supplementary table 4: Summary of candidate genes within the Chr 8 QTL. Gene names and their mm10 start locations are shown, along with phenotypes of interest from the Mouse Genome Informatics database and NCBI GeneRIFs and Pubmed abstracts. Presence of gene expression in the B6 postnatal day 4 brain from the Allen brain atlas developing brain map is shown (RSP = rostral secondary prosencephalon, tel = telencephalic vesicle, PHy = peduncular (caudal) hypothalamus, p3 = prosomere 3, p2 = prosomere 2, p1 = prosomere 1, M = midbrain, PPH = prepontine hindbrain, PH = pontine hindbrain, PMH = pontomedullary hindbrain, MH = medullary hindbrain). Gene expression data in the BXD family from GeneNetwork was used to identify brain regions with significant *cis-*eQTL and where there were correlations between gene expression and our measured traits of interest (brain regions used were postnatal day 3 neocortex, postnatal day 3 striatum, postnatal day 14 neocortex, postnatal day 14 striatum, adult amygdala, adult cerebellum, adult hippocampal precursor cells, adult hippocampus, adult hypothalamous, adult midbrain, adult neocortex, adult nucleus accumbens, adult prefrontal cortex, adult striatum, adult ventral tegmental area: PN = postnatal). Correlations were calculated within GeneNetwork using the Spearman correlation coefficient, ρ.

**References for supplementary materials**

Al-Shami, A., Jhaver, K.G., Vogel, P., Wilkins, C., Humphries, J., Davis, J.J., Xu, N., Potter, D.G., Gerhardt, B., Mullinax, R., Shirley, C.R., Anderson, S.J. & Oravecz, T. (2010) Regulators of the proteasome pathway, Uch37 and Rpn13, play distinct roles in mouse development. *PLoS One* **5**, e13654.

Ashbrook, D.G., Gini, B. & Hager, R. (2015) Genetic variation in offspring indirectly influences the quality of maternal behaviour in mice. *Elife* **4**, e11814.

Ashbrook, D.G., Sharmin, N. & Hager, R. (2017) Offspring genes indirectly influence sibling and maternal beha vioural strategies over resource share. *Proc R Soc B Biol Sci* **284**, 20171059.

Bartlett, C.W., Flax, J.F., Logue, M.W., Smith, B.J., Vieland, V.J., Tallal, P. & Brzustowicz, L.M. (2004) Examination of potential overlap in autism and language loci on chromosomes 2, 7, and 13 in two independent samples ascertained for specific language impairment. *Hum Hered* **57**, 10–20.

Bartlett, C.W., Flax, J.F., Logue, M.W., Vieland, V.J., Bassett, A.S., Tallal, P. & Brzustowicz, L.M. (2002) A major susceptibility locus for specific language impairment is located on 13q21. *Am J Hum Genet* **71**, 45–55.

Bolivar, V.J., Scott Ganus, J. & Messer, A. (2002) The development of behavioral abnormalities in the motor neuron degeneration (mnd) mouse. *Brain Res* **937**, 74–82.

Bradford, Y., Haines, J., Hutcheson, H., Gardiner, M., Braun, T., Sheffield, V., Cassavant, T., Huang, W., Wang, K., Vieland, V., Folstein, S., Santangelo, S. & Piven, J. (2001) Incorporating language phenotypes strengthens evidence of linkage to autism. *Am J Med Genet* **105**, 539–547.

Cohen-Salmon, C., Carlier, M., Roubertoux, P., Jouhaneau, J., Semal, C. & Paillette, M. (1985) Differences in patterns of pup care in mice. V--Pup ultrasonic emissions and pup care behavior. *Physiol Behav* **35**, 167–174.

D’Amato, F.R. & Populin, R. (1987) Mother-offspring interaction and pup development in genetically deaf mice. *Behav Genet* **17**, 465–475.

Do, D.N., Strathe, A.B., Ostersen, T., Jensen, J., Mark, T. & Kadarmideen, H.N. (2013) Genome-wide association study reveals genetic architecture of eating behavior in pigs and its implications for humans obesity by comparative mapping. *PLoS One* **8**, e71509.

Ehret, G. & Haack, B. (1982) Ultrasound recognition in house mice: Key-Stimulus configuration and recognition mechanism. *J Comp Physiol ? A* **148**, 245–251.

Fitzpatrick, K., Winrow, C.J., Gotter, A.L., Millstein, J., Arbuzova, J., Brunner, J., Kasarskis, A., Vitaterna, M.H., Renger, J.J. & Turek, F.W. (2012) Altered sleep and affect in the neurotensin receptor 1 knockout mouse. *Sleep* **35**, 949–956.

Li, J.-M., Lu, C.-L., Cheng, M.-C., Luu, S.-U., Hsu, S.-H., Hu, T.-M., Tsai, H.-Y. & Chen, C.-H. (2014) Role of the DLGAP2 gene encoding the SAP90/PSD-95-associated protein 2 in schizophrenia. *PLoS One* **9**, e85373.

Nakagawa, R., Matsunaga, E. & Okanoya, K. (2012) Defects in ultrasonic vocalization of cadherin-6 knockout mice. *PLoS One* **7**, e49233.

Noirot, E. (1964) Changes in responsiveness to young in the adult mouse: The effect of external stimuli. *J Comp Physiol Psychol* **57**, 97–99.

Okon, E.E. (1970) The effect of environmental temperature on the production of ultrasounds by isolated non-handled albino mouse pups. *J Zool* **162**, 71–83.

Patten, E., Belardi, K., Baranek, G.T., Watson, L.R., Labban, J.D. & Oller, D.K. (2014) Vocal patterns in infants with autism spectrum disorder: canonical babbling status and vocalization frequency. *J Autism Dev Disord* **44**, 2413–2428.

Pinto, D., Pagnamenta, A.T., Klei, L., Anney, R., Merico, D., Regan, R., Conroy, J., Magalhaes, T.R., Correia, C., Abrahams, B.S., Almeida, J., Bacchelli, E., Bader, G.D., Bailey, A.J., Baird, G., Battaglia, A., Berney, T., Bolshakova, N., Bölte, S., Bolton, P.F., Bourgeron, T., Brennan, S., Brian, J., Bryson, S.E., Carson, A.R., Casallo, G., Casey, J., Chung, B.H.Y., Cochrane, L., Corsello, C., Crawford, E.L., Crossett, A., Cytrynbaum, C., Dawson, G., de Jonge, M., Delorme, R., Drmic, I., Duketis, E., Duque, F., Estes, A., Farrar, P., Fernandez, B.A., Folstein, S.E., Fombonne, E., Freitag, C.M., Gilbert, J., Gillberg, C., Glessner, J.T., Goldberg, J., Green, A., Green, J., Guter, S.J., Hakonarson, H., Heron, E.A., Hill, M., Holt, R., Howe, J.L., Hughes, G., Hus, V., Igliozzi, R., Kim, C., Klauck, S.M., Kolevzon, A., Korvatska, O., Kustanovich, V., Lajonchere, C.M., Lamb, J.A., Laskawiec, M., Leboyer, M., Le Couteur, A., Leventhal, B.L., Lionel, A.C., Liu, X.-Q., Lord, C., Lotspeich, L., Lund, S.C., Maestrini, E., Mahoney, W., Mantoulan, C., Marshall, C.R., McConachie, H., McDougle, C.J., McGrath, J., McMahon, W.M., Merikangas, A., Migita, O., Minshew, N.J., Mirza, G.K., Munson, J., Nelson, S.F., Noakes, C., Noor, A., Nygren, G., Oliveira, G., Papanikolaou, K., Parr, J.R., Parrini, B., Paton, T., Pickles, A., Pilorge, M., Piven, J., Ponting, C.P., Posey, D.J., Poustka, A., Poustka, F., Prasad, A., Ragoussis, J., Renshaw, K., Rickaby, J., Roberts, W., Roeder, K., Roge, B., Rutter, M.L., Bierut, L.J., Rice, J.P., Salt, J., Sansom, K., Sato, D., Segurado, R., Sequeira, A.F., Senman, L., Shah, N., Sheffield, V.C., Soorya, L., Sousa, I., Stein, O., Sykes, N., Stoppioni, V., Strawbridge, C., Tancredi, R., Tansey, K., Thiruvahindrapduram, B., Thompson, A.P., Thomson, S., Tryfon, A., Tsiantis, J., Van Engeland, H., Vincent, J.B., Volkmar, F., Wallace, S., Wang, K., Wang, Z., Wassink, T.H., Webber, C., Weksberg, R., Wing, K., Wittemeyer, K., Wood, S., Wu, J., Yaspan, B.L., Zurawiecki, D., Zwaigenbaum, L., Buxbaum, J.D., Cantor, R.M., Cook, E.H., Coon, H., Cuccaro, M.L., Devlin, B., Ennis, S., Gallagher, L., Geschwind, D.H., Gill, M., Haines, J.L., Hallmayer, J., Miller, J., Monaco, A.P., Nurnberger, J.I., Paterson, A.D., Pericak-Vance, M.A., Schellenberg, G.D., Szatmari, P., Vicente, A.M., Vieland, V.J., Wijsman, E.M., Scherer, S.W., Sutcliffe, J.S. & Betancur, C. (2010) Functional impact of global rare copy number variation in autism spectrum disorders. *Nature* **466**, 368–372.

Remaury, A., Vita, N., Gendreau, S., Jung, M., Arnone, M., Poncelet, M., Culouscou, J.-M., Le Fur, G., Soubrié, P., Caput, D., Shire, D., Kopf, M. & Ferrara, P. (2002) Targeted inactivation of the neurotensin type 1 receptor reveals its role in body temperature control and feeding behavior but not in analgesia. *Brain Res* **953**, 63–72.

Scearce-Levie, K., Roberson, E.D., Gerstein, H., Cholfin, J.A., Mandiyan, V.S., Shah, N.M., Rubenstein, J.L.R. & Mucke, L. (2008) Abnormal social behaviors in mice lacking Fgf17. *Genes Brain Behav* **7**, 344–354.

Smith, J.C. (1976) Responses of adult mice to models of infant calls. *J Comp Physiol Psychol* **90**, 1105–1115.

Smith, M., Woodroffe, A., Smith, R., Holguin, S., Martinez, J., Filipek, P.A., Modahl, C., Moore, B., Bocian, M.E., Mays, L., Laulhere, T., Flodman, P. & Spence, M.A. (2002) Molecular genetic delineation of a deletion of chromosome 13q12-->q13 in a patient with autism and auditory processing deficits. *Cytogenet Genome Res* **98**, 233–239.

Steele, F.F., Whitehouse, S.C., Aday, J.S. & Prus, A.J. (2017) Neurotensin NTS1 and NTS2 receptor agonists produce anxiolytic-like effects in the 22-kHz ultrasonic vocalization model in rats. *Brain Res* **1658**, 31–35.

Steen, V.M., Nepal, C., Ersland, K.M., Holdhus, R., Nævdal, M., Ratvik, S.M., Skrede, S. & Håvik, B. (2013) Neuropsychological deficits in mice depleted of the schizophrenia susceptibility gene CSMD1. *PLoS One* **8**, e79501.

Thoenes, M., Zimmermann, U., Ebermann, I., Ptok, M., Lewis, M.A., Thiele, H., Morlot, S., Hess, M.M., Gal, A., Eisenberger, T., Bergmann, C., Nürnberg, G., Nürnberg, P., Steel, K.P., Knipper, M. & Bolz, H.J. (2015) OSBPL2 encodes a protein of inner and outer hair cell stereocilia and is mutated in autosomal dominant hearing loss (DFNA67). *Orphanet J Rare Dis* **10**, 15.

Truong, D.T., Shriberg, L.D., Smith, S.D., Chapman, K.L., Scheer-Cohen, A.R., DeMille, M.M.C., Adams, A.K., Nato, A.Q., Wijsman, E.M., Eicher, J.D. & Gruen, J.R. (2016) Multipoint genome-wide linkage scan for nonword repetition in a multigenerational family further supports chromosome 13q as a locus for verbal trait disorders. *Hum Genet* **135**, 1329–1341.

Tylee, D.S., Espinoza, A.J., Hess, J.L., Tahir, M.A., McCoy, S.Y., Rim, J.K., Dhimal, T., Cohen, O.S. & Glatt, S.J. (2017) RNA sequencing of transformed lymphoblastoid cells from siblings discordant for autism spectrum disorders reveals transcriptomic and functional alterations: Evidence for sex-specific effects. *Autism Res* **10**, 439–455.

Xing, G., Yao, J., Wu, B., Liu, T., Wei, Q., Liu, C., Lu, Y., Chen, Z., Zheng, H., Yang, X. & Cao, X. (2015) Identification of OSBPL2 as a novel candidate gene for progressive nonsyndromic hearing loss by whole-exome sequencing. *Genet Med* **17**, 210–218.

Xu, D., Gilkerson, J., Richards, J., Yapanel, U. & Gray, S. (2009) Child vocalization composition as discriminant information for automatic autism detection. *Conf Proc . Annu Int Conf IEEE Eng Med Biol Soc IEEE Eng Med Biol Soc Annu Conf* **2009**, 2518–2522.
