# Supplementary figures and images for: Born to Cry: A Genetic Dissection of Infant Vocalization
Source: Front Behav Neurosci. 2018 Oct 29;12:250. doi: 10.3389/fnbeh.2018.00250 (PMC6216097; doi:10.3389/fnbeh.2018.00250)

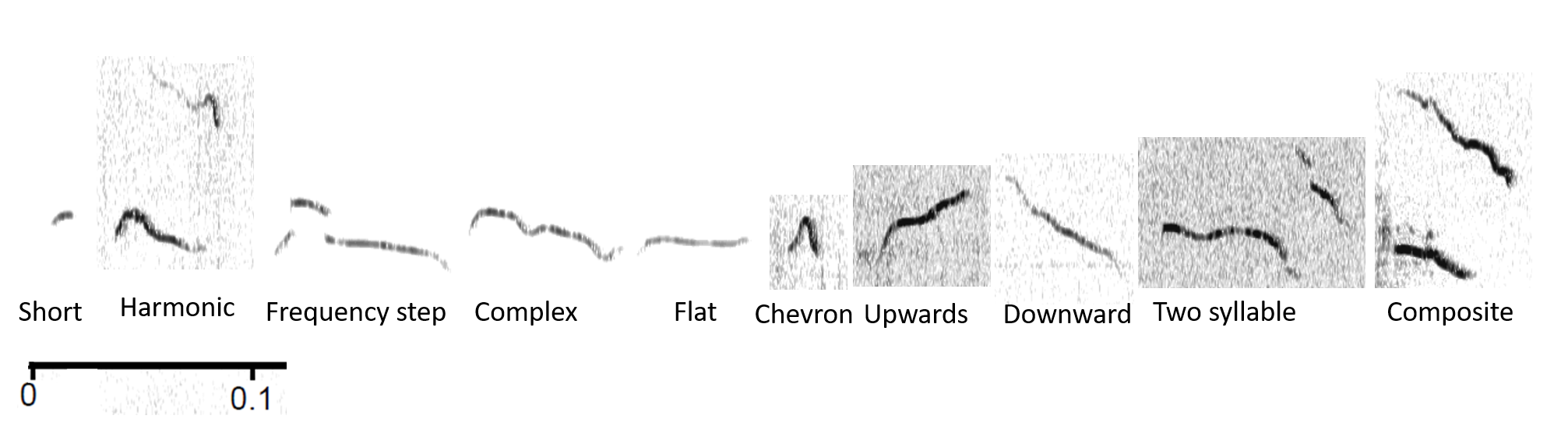

Supplement: Supplementary file 6 [file Image_1.TIF]

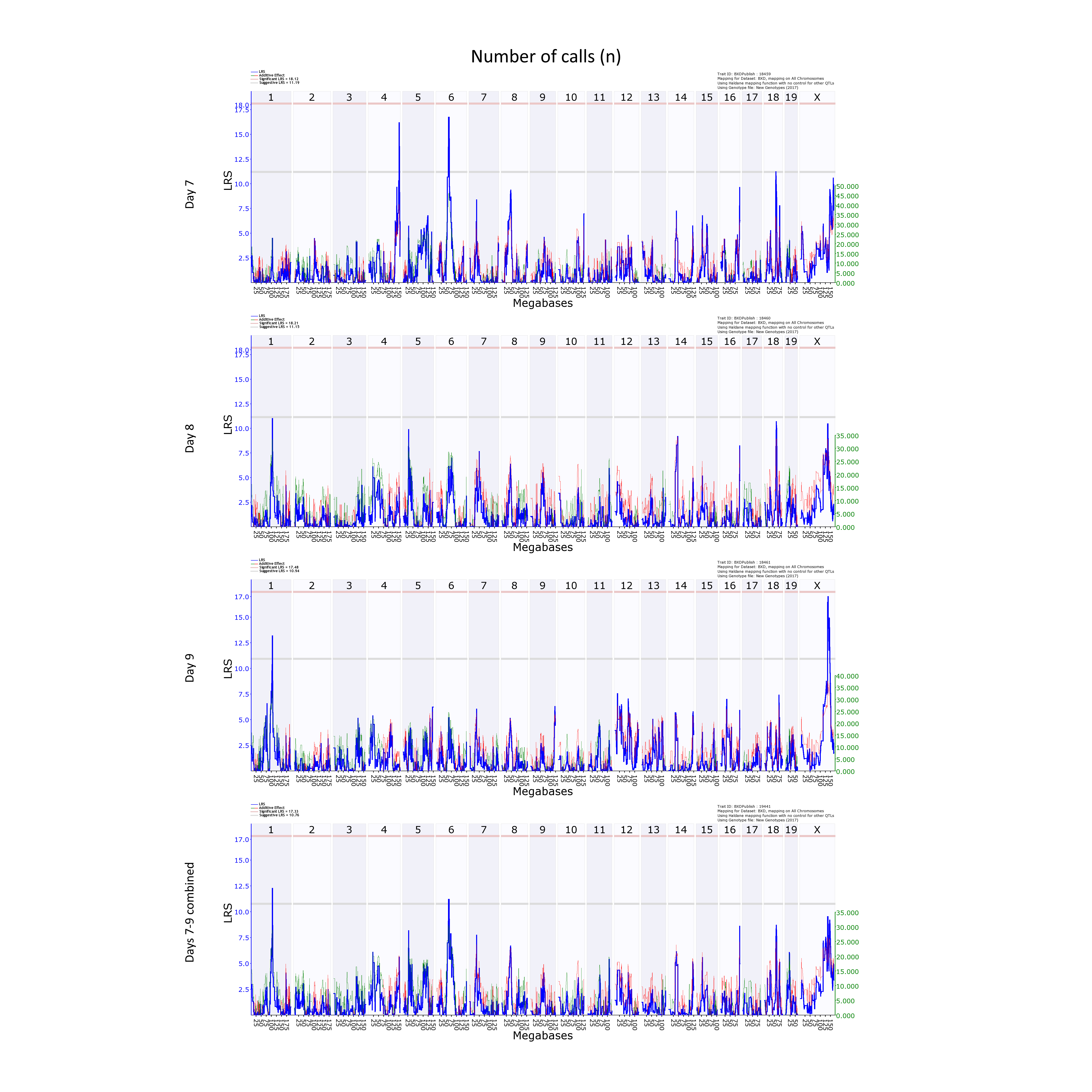

Supplement: Supplementary file 7 [file Image_2.TIF]

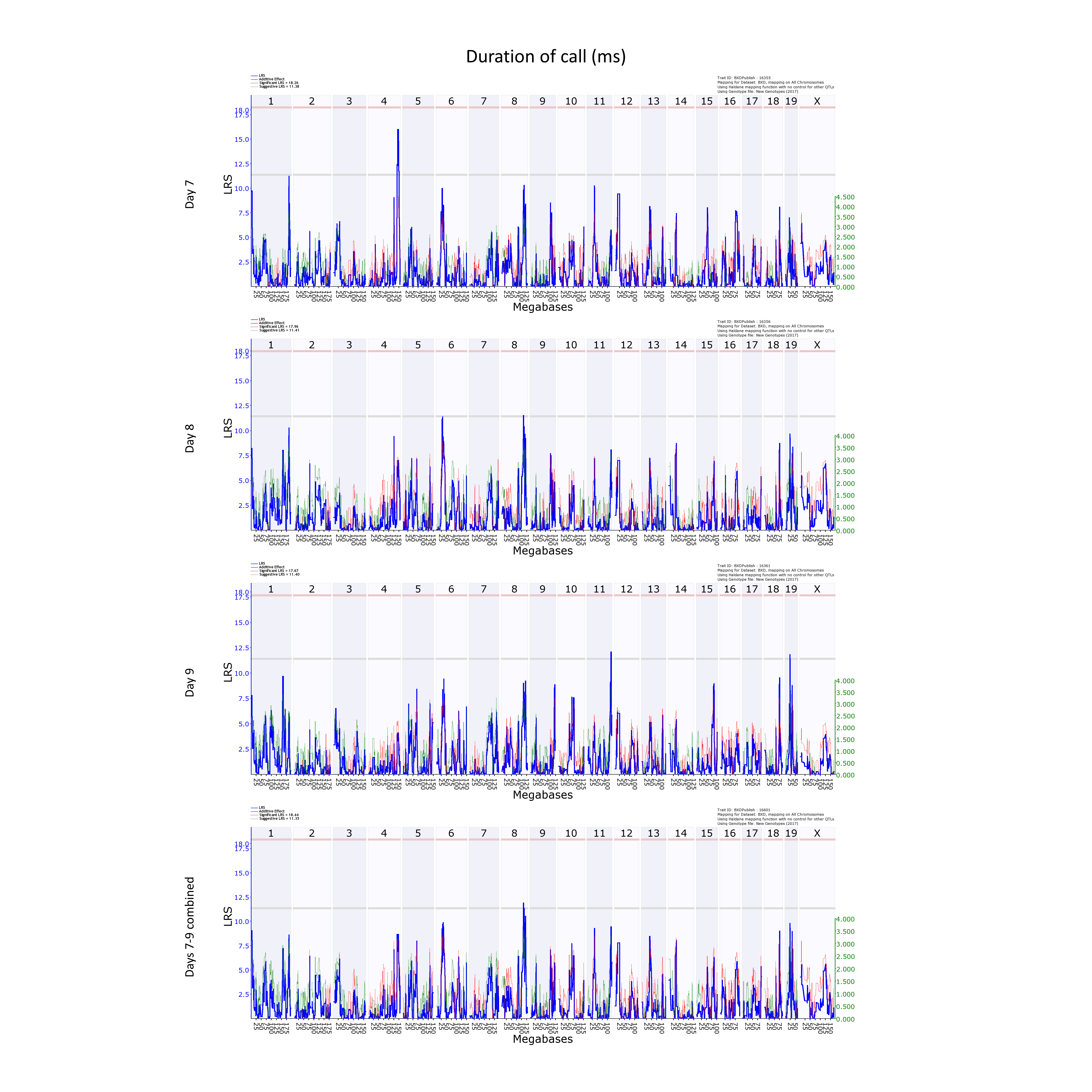

Supplement: Supplementary file 8 [file Image_3.TIF]

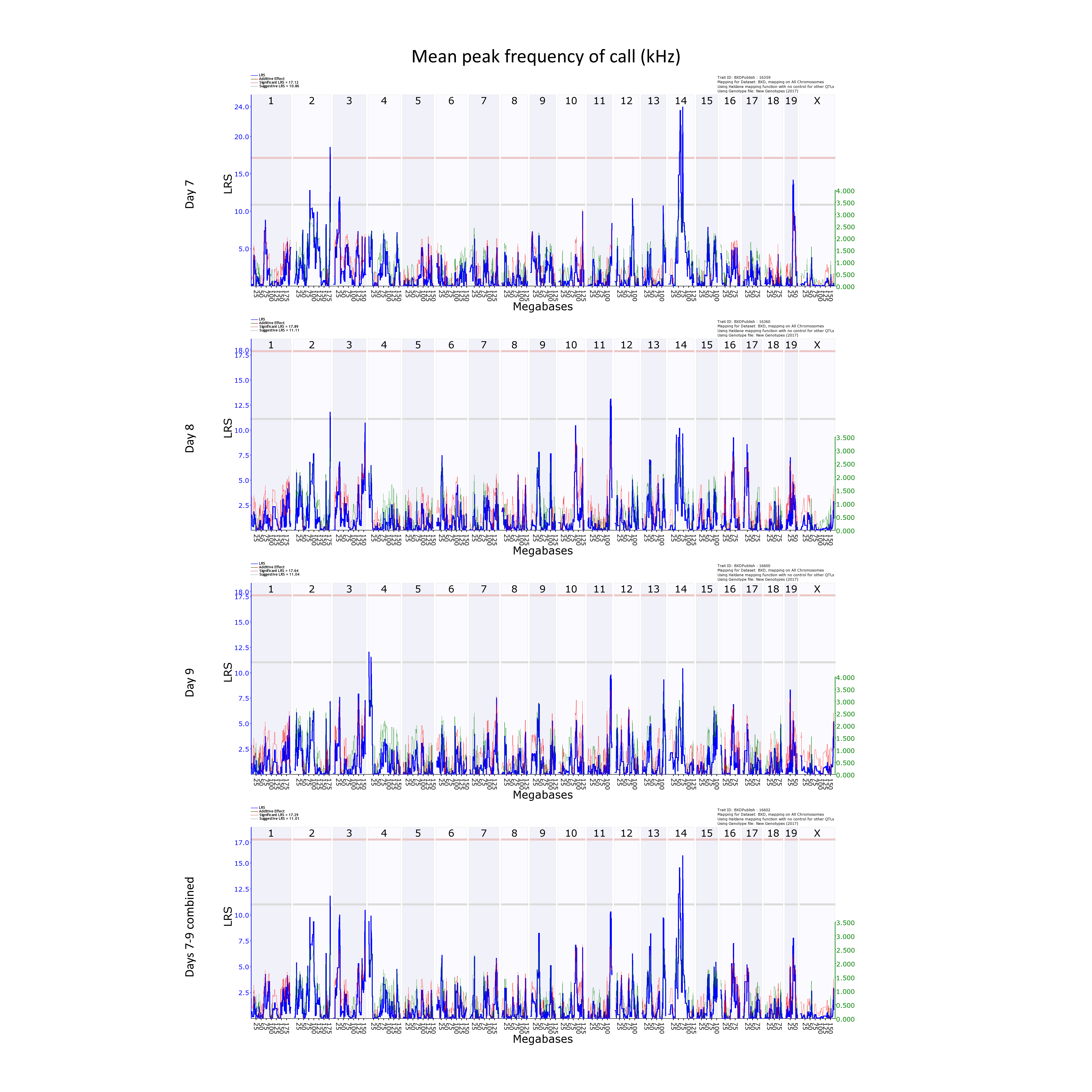

Supplement: Supplementary file 9 [file Image_4.TIF]

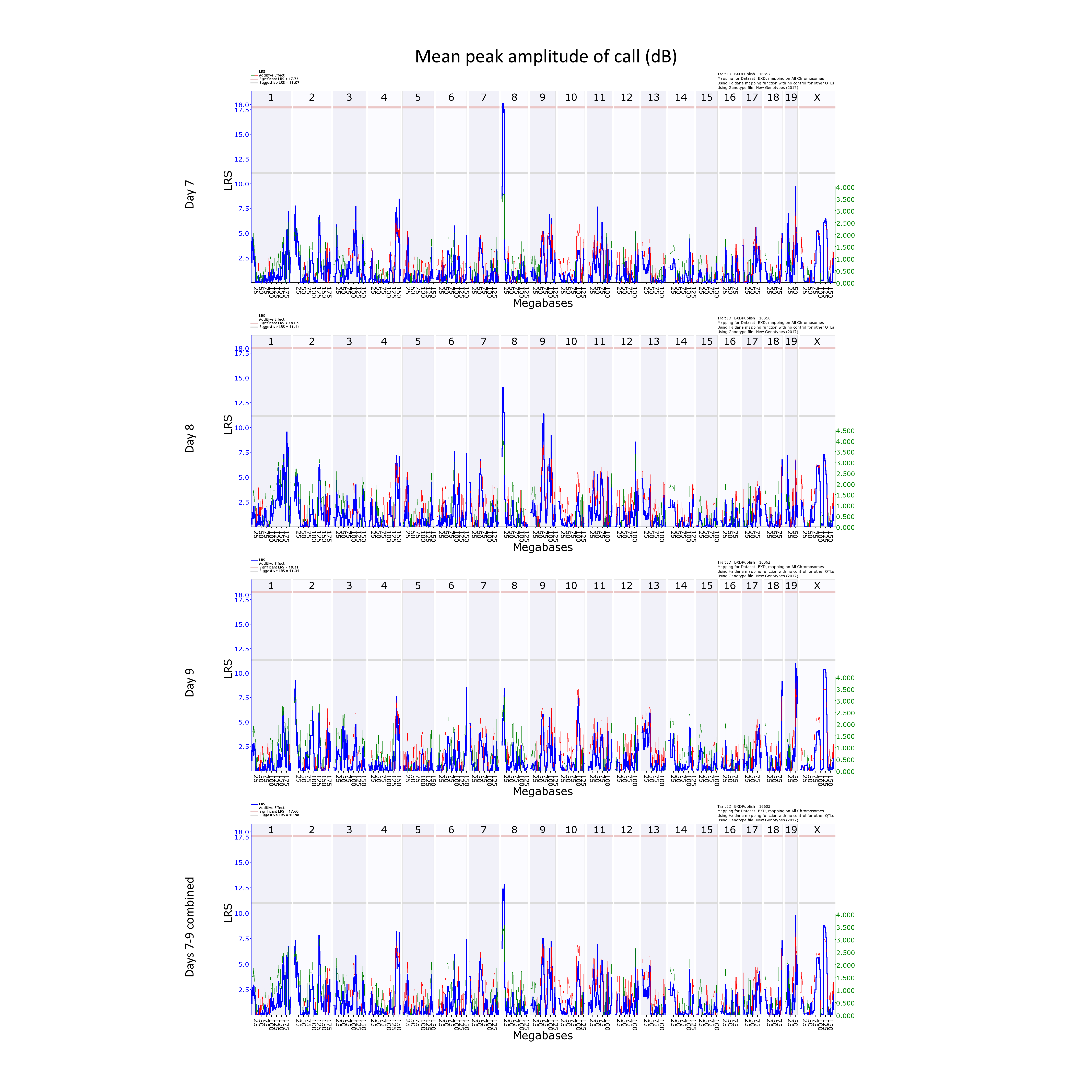

Supplement: Supplementary file 10 [file Image_5.TIF]

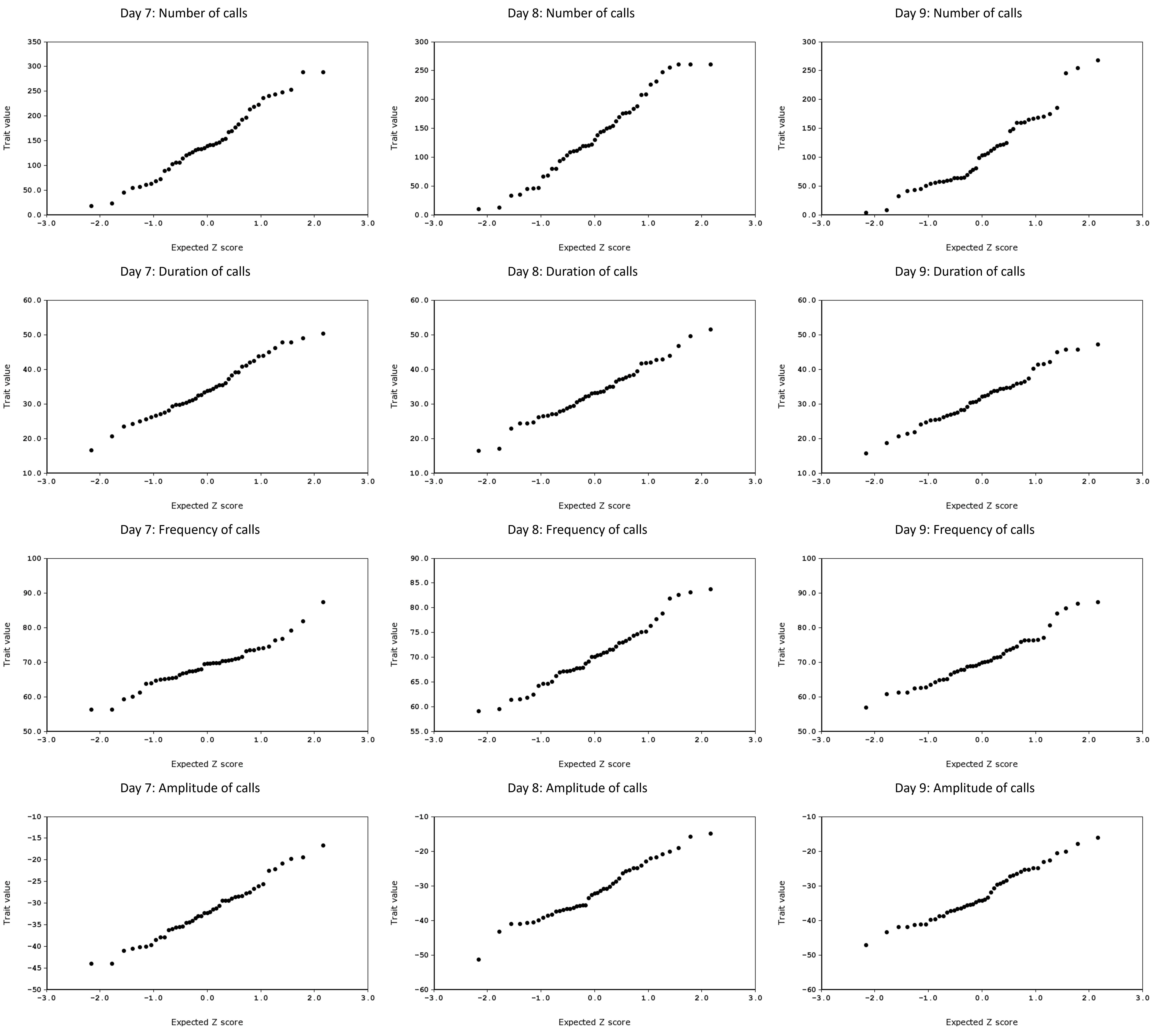

Supplement: Supplementary file 11 [file Image_6.TIF]

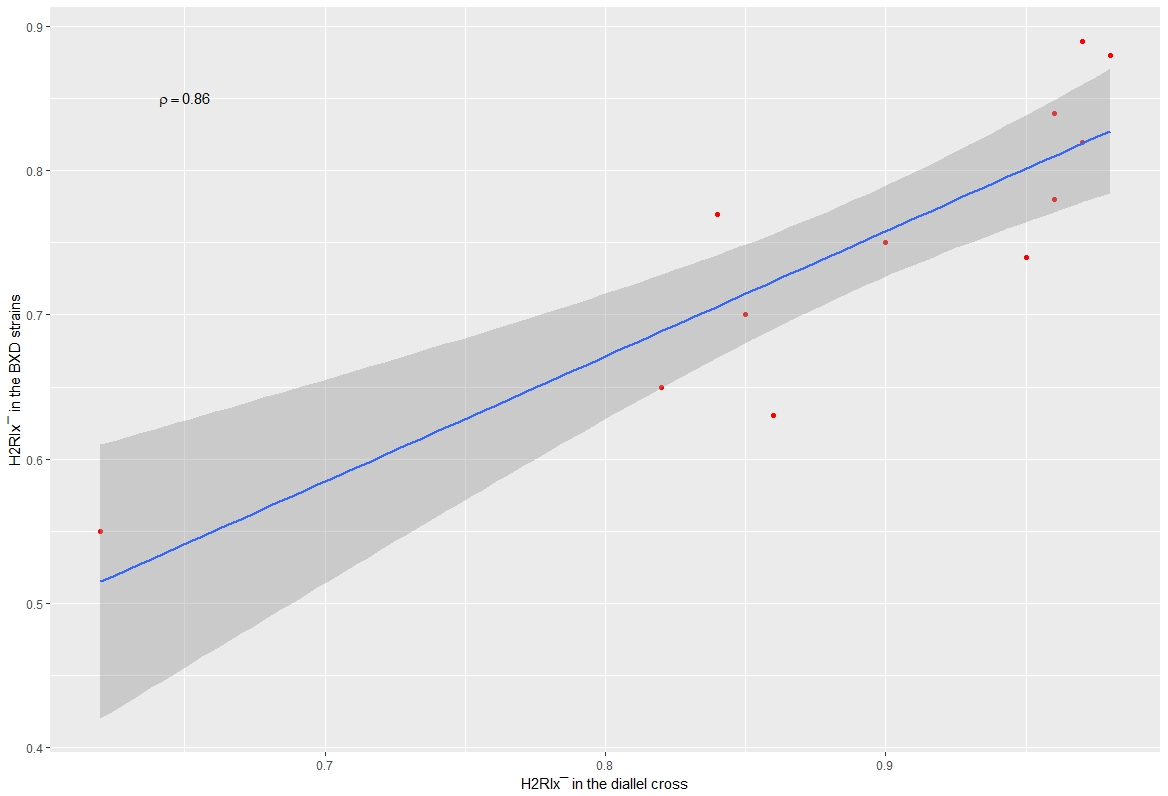

Supplement: Supplementary file 12 [file Image_7.JPEG]

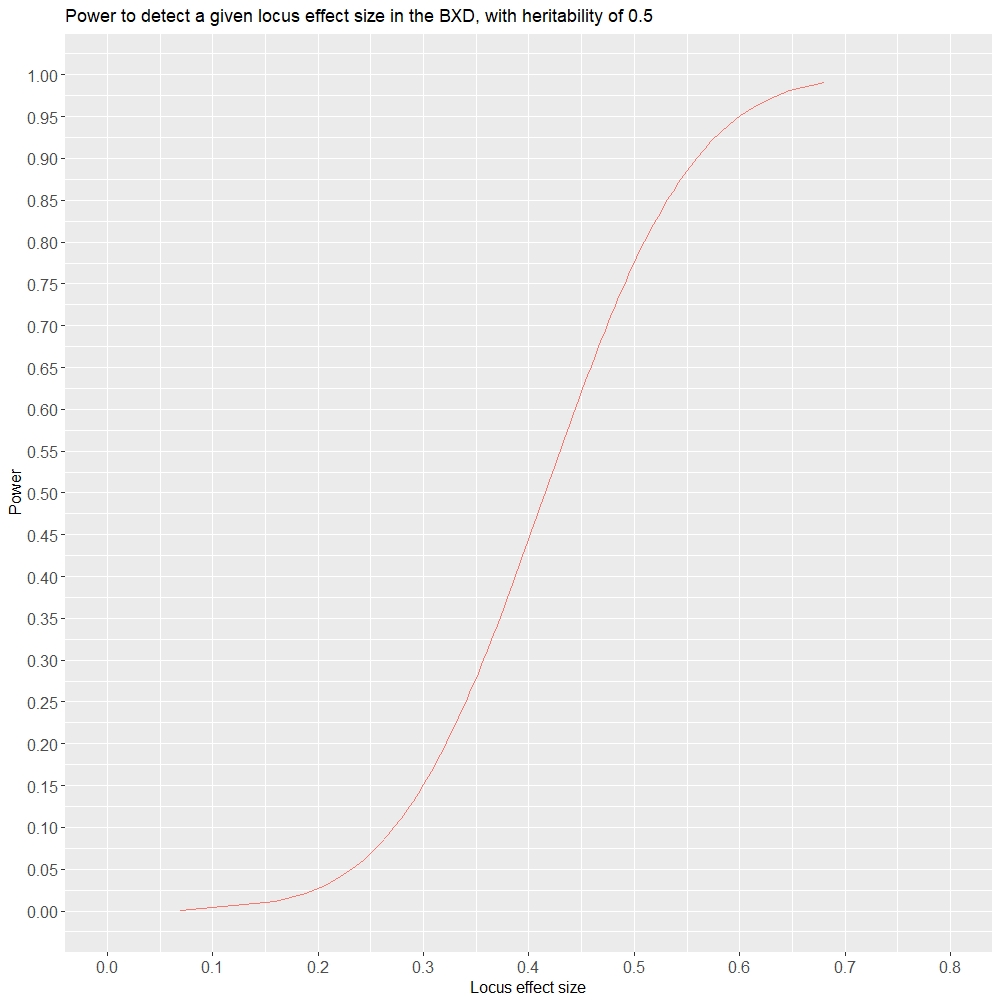

Supplement: Supplementary file 13 [file Image_8.JPEG]

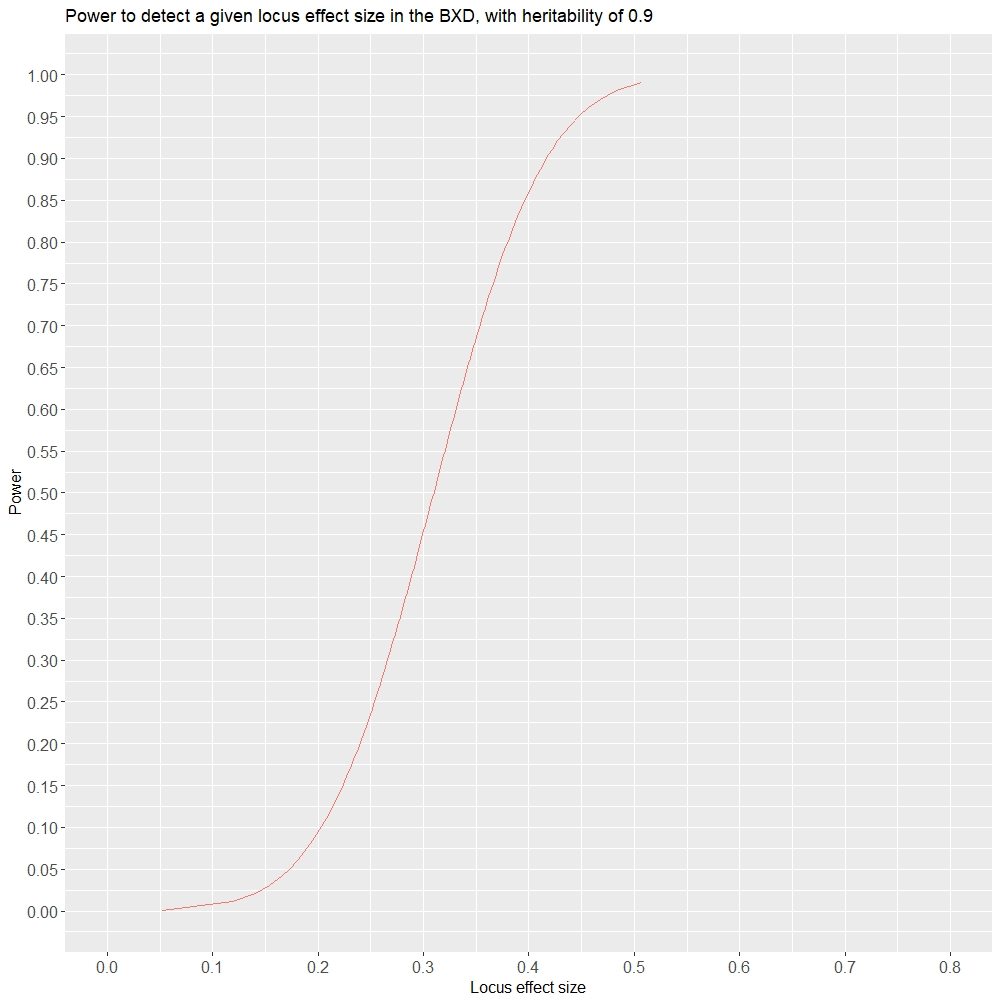

Supplement: Supplementary file 14 [file Image_9.JPEG]

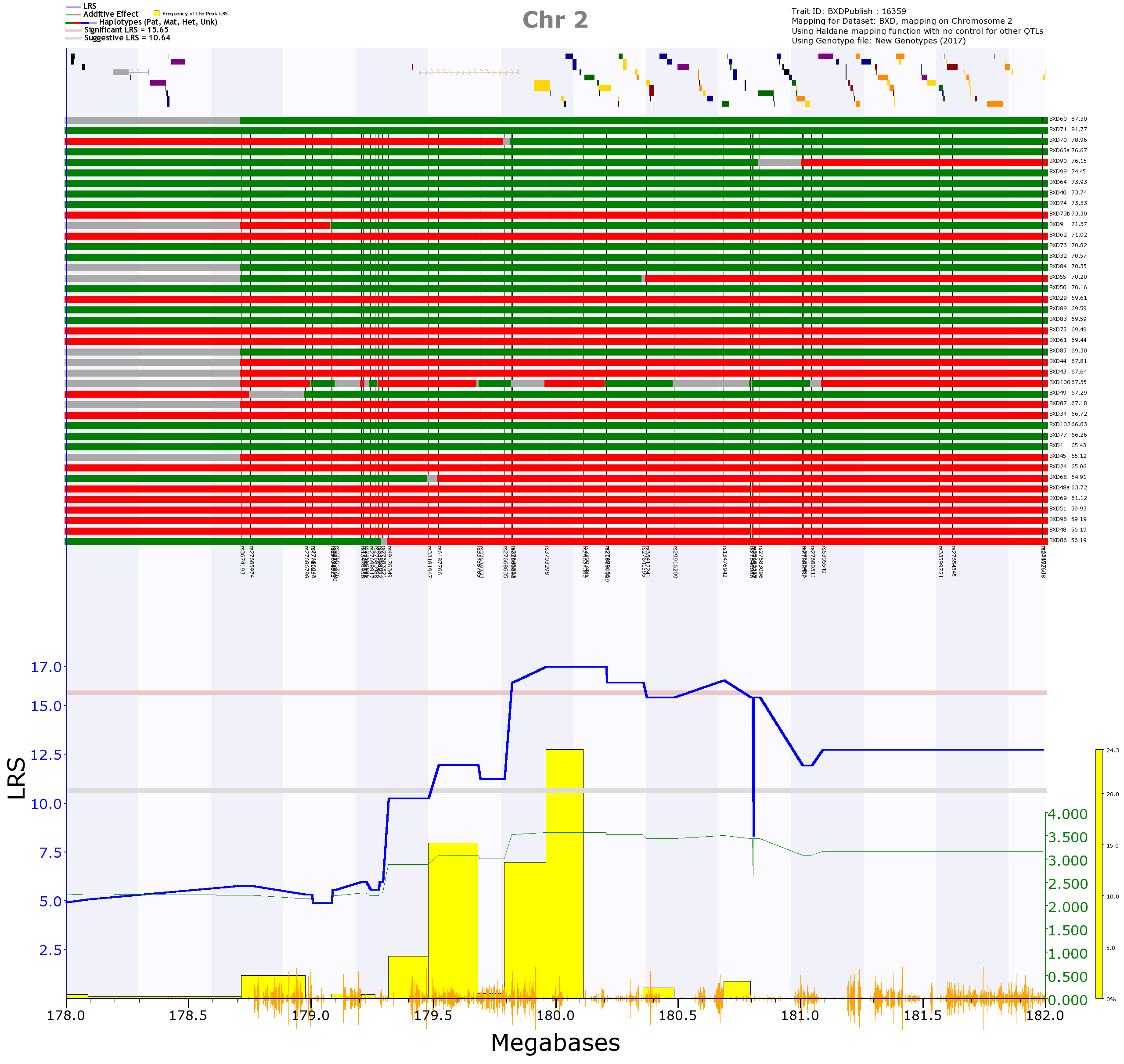

Supplement: Supplementary file 15 [file Image_10.TIFF]

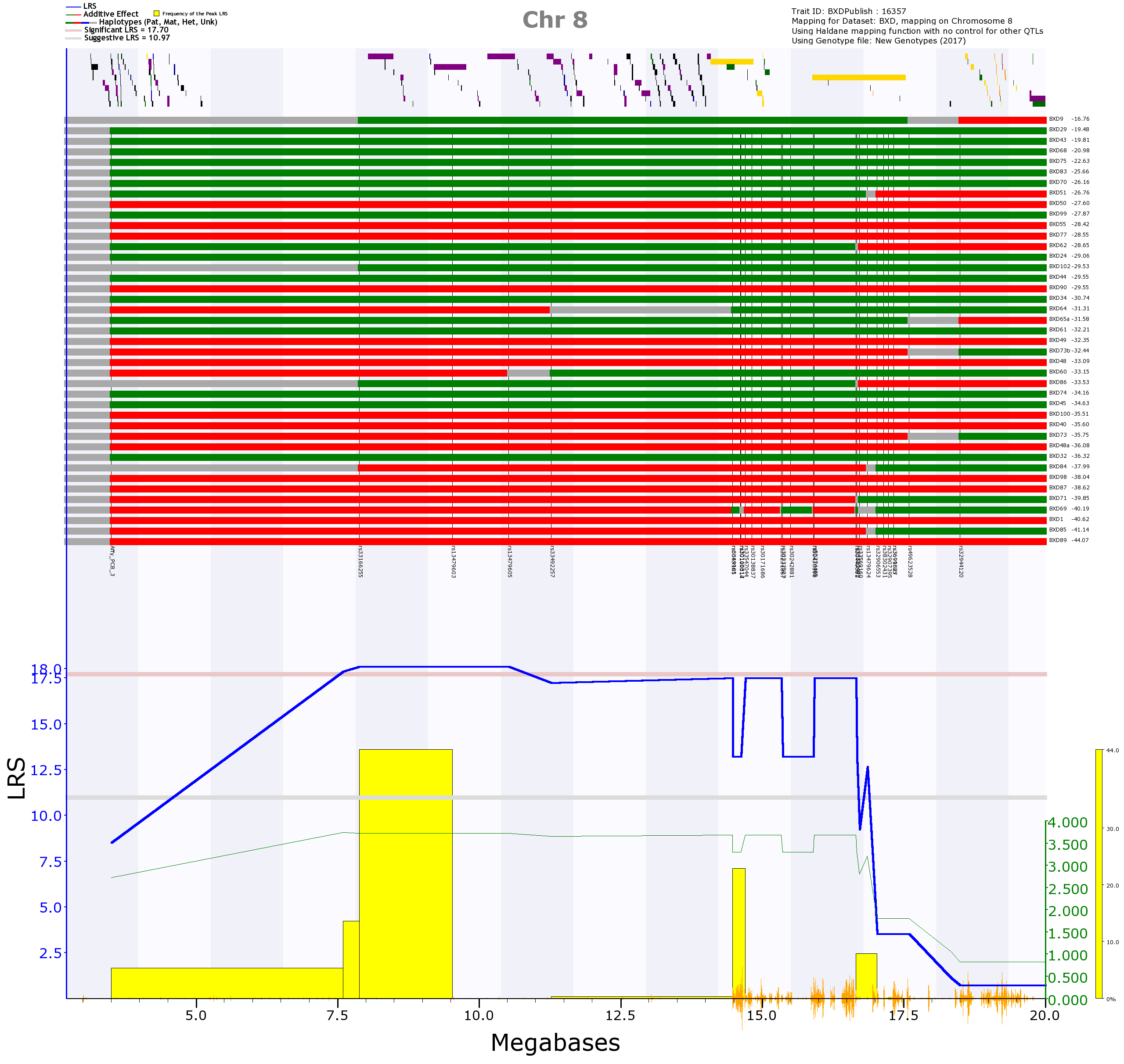

Supplement: Supplementary file 16 [file Image_11.TIFF]
